# Unraveling Protein Interactions between the Temperate Virus Bam35 and Its *Bacillus* Host Using an Integrative Yeast Two Hybrid–High Throughput Sequencing Approach

**DOI:** 10.3390/ijms222011105

**Published:** 2021-10-14

**Authors:** Ana Lechuga, Cédric Lood, Mónica Berjón-Otero, Alicia del Prado, Jeroen Wagemans, Vera van Noort, Rob Lavigne, Margarita Salas, Modesto Redrejo-Rodríguez

**Affiliations:** 1 Centro de Biología Molecular Severo Ochoa (CSIC-UAM), 28049 Madrid, Spain; ana.lechuga@kuleuven.be (A.L.); monicaberjon@hotmail.com (M.B.-O.); a.delprado@csic.es (A.d.P.); msalas@cbm.csic.es (M.S.); 2 Laboratory of Gene Technology, Department of Biosystems, KU Leuven, 3001 Leuven, Belgium; cedric.lood@kuleuven.be (C.L.); jeroen.wagemans@kuleuven.be (J.W.); rob.lavigne@kuleuven.be (R.L.); 3 Centre of Microbial and Plant Genetics, Laboratory of Computational Systems Biology, Department of Microbial and Molecular Systems, KU Leuven, 3001 Leuven, Belgium; vera.vannoort@kuleuven.be; 4 Departamento de Bioquímica, Universidad Autónoma de Madrid (UAM) and Instituto de Investigaciones Biomédicas Alberto Sols (CSIC-UAM), 28029 Madrid, Spain

**Keywords:** Bam35, tectivirus, *Betatectivirus*, interactome, yeast two-hybrid, *Bacillus thuringiensis*, protein–protein interactions, high-throughput sequencing

## Abstract

*Bacillus virus Bam35* is the model *Betatectivirus* and member of the family *Tectiviridae*, which is composed of tailless, icosahedral, and membrane-containing bacteriophages. Interest in these viruses has greatly increased in recent years as they are thought to be an evolutionary link between diverse groups of prokaryotic and eukaryotic viruses. Additionally, betatectiviruses infect bacteria of the *Bacillus cereus* group, which are known for their applications in industry and notorious since it contains many pathogens. Here, we present the first protein–protein interactions (PPIs) network for a tectivirus–host system by studying the Bam35–*Bacillus thuringiensis* model using a novel approach that integrates the traditional yeast two-hybrid system and high-throughput sequencing (Y2H-HTS). We generated and thoroughly analyzed a genomic library of Bam35′s host *B.* *thuringiensis* HER1410 and screened interactions with all the viral proteins using different combinations of bait–prey couples. Initial analysis of the raw data enabled the identification of over 4000 candidate interactions, which were sequentially filtered to produce 182 high-confidence interactions that were defined as part of the core virus–host interactome. Overall, host metabolism proteins and peptidases were particularly enriched within the detected interactions, distinguishing this host–phage system from the other reported host–phage PPIs. Our approach also suggested biological roles for several Bam35 proteins of unknown function, including the membrane structural protein P25, which may be a viral hub with a role in host membrane modification during viral particle morphogenesis. This work resulted in a better understanding of the Bam35–*B. thuringiensis* interaction at the molecular level and holds great potential for the generalization of the Y2H-HTS approach for other virus–host models.

## 1. Introduction

The family *Tectiviridae* is defined as a family of tailless, icosahedral viruses with a lipidic inner membrane and a linear, double-stranded DNA genome of approximately 15 kb, which is capped by the so-called terminal protein [1]. These phages are currently divided into five genera, encompassing lytic and lysogenic bacteriophages that infect Gram-negative or Gram-positive bacteria, respectively. The early and best-known lytic viruses that prey on Gram-negative bacteria belong to the *Alphatectivirus* genus, whereas the temperate phages infecting Gram-positive bacteria were grouped in the *Betatectivirus* genus, and the recently reported new members of the family were assigned to the *Gamma*-, *Delta*-, and *Epsilontectivirus* genera [2]. This family spans a wide genetic diversity under a common morphology with a broad host range and a predicted significant ecological importance [3,4]. Additionally, they were proposed to be related to the origin of some mobile elements and groups of eukaryotic DNA viruses [5,6]. Among the *Tectiviridae* members, the interest in betatectiviruses has increased in the last decade due to their ability to infect different members of the *Bacillus cereus* group [7]. Indeed, some specifically infect pathogenic bacteria, such as phages Wip1 and AP50, can infect *Bacillus anthracis*, which is the etiological agent of anthrax [8,9], or Sole and Simila, which infects the food pathogen *B. cereus* [10]. 

The model virus for molecular and structural studies on betatectiviruses is *Bacillus virus Bam35* [11,12,13,14]. This phage infects *Bacillus thuringiensis*, which is a type species of the *B. cereus* group that is known for its entomocidal capacity and is broadly used as biopesticide for pest control [15,16]. The betatectivirus genome organization is modular and traditionally segmented into three functional categories (Supplementary Figure S1): (i) gene regulation and genome replication, (ii) virion structure and DNA packaging, and (iii) host recognition and cell lysis [17]. Despite the conserved genome organization between Bam35 and the widely characterized model virus for *Alphatectivirus* phage PRD1, there is a very low sequence identity between them. Moreover, the limited similarity to proteins that are published in databases hinders the functional annotation of Bam35 and other tectiviruses. This problem was addressed by comparative studies, single protein purification and analysis, and protein–protein interaction (PPI) studies, resulting in the current functional annotation of 23 out of the 32 open reading frames of Bam35 [11,13,18,19]. 

The icosahedral capsid of Bam35 is mainly composed of the major capsid proteins that form the facets, and the penton proteins that are located in the eleven vertices and incorporate the flexible spikes. Packaging and injection of DNA take place through the 12th vertex, which is also known as the special vertex [12]. Both capsid proteins and dsDNA interact with inner membrane lipids [12,20]. Although not all membrane proteins have been identified for Bam35, P25 is probably a membrane structural component and P26 is a conserved transglycosylase that seems to be a cornerstone transmembrane protein that interacts with both lytic and capsid proteins [19,21]. 

Bam35 was proposed to infect host cells following a three-step mechanism. First, the flexible spikes recognize and bind the cell surface receptor. So far, only one of the components of this receptor has been identified, namely, the N-acetyl-muramic acid, which is essential for phage adsorption [22]. Second, the peptidoglycan hydrolyzing proteins facilitate overcoming the cell wall barrier to access the plasma membrane. Finally, as in PRD1, Bam35 forms a tail-like structure that consists of a proteo-lipidic tube that protrudes from the inner lipid membrane and delivers the linear dsDNA into the cell [12,22,23]. The Bam35 genome is replicated by a protein-primed mechanism that uses a terminal protein (TP) to prime the genome replication and thus remains linked to the 5′ DNA ends [13,24,25]. Upon infection of the cell, this temperate phage can establish a lysogenic state as a linear episome. The lysis–lysogeny switch was studied regarding the GIL01 virus, which is almost identical to Bam35 [26]. During lysogeny, the host transcription repressor LexA remains bound to viral protein P7 and restricts the transcription of the late genes [18,27,28]. However, only low-expression levels were suggested for all viral genes during the GIL01/Bam35 temperate phase. Nonetheless, the phage impacts bacterial growth, sporulation, motility, and biofilm formation [29]. Host cell activation of the SOS response allows the phage to enter the lytic cycle, which is mediated by the elimination of transcription repression and transcription activation of the late genes by the viral protein P6 [30]. In a final step during the lytic cycle, Bam35 virions are released via lysis, likely through an endolysin–holin system [31]. Although two endolysins were described (P26 and P30), no holin has been identified to date [21]. 

The protein intraviral interactome of Bam35, as well as of other viruses, recently revealed new functions and the localization of phage ORFan proteins [19]. However, understanding host–virus PPIs is also essential for studying protein functions, life cycle, and evolution, and is particularly helpful for identifying molecular targets to combat pathogens [32]. The last decade has seen the emergence of “omics” approaches as investigative tools for the study of biological pathways that are involved in pathogen replication, host response, and, eventually, infection progression. Among the methods that are used in high-throughput interactomics, the yeast two-hybrid system (Y2H) remains one of the most widely used techniques for studying PPIs [33]. Some of the first Y2H studies involving viruses addressed the interaction between bacteria and their phages. These included *Escherichia coli* phages T7 and λ, *Pseudomonas aeruginosa* phages, *Streptococcus pneumoniae* phages Cp-1 and Dp-1, and mycobacteriophage Giles [34,35,36,37,38,39]. Other techniques for detecting PPIs, such as affinity purification coupled to mass spectrometry, also significantly contributed to the study of phage–bacteria interactions [40,41]. These works suggest different and specialized PPI networks, reflecting their genetic diversity, distinct biology, and diverging co-evolution with their specific hosts [42]. Despite phage–host specialization, proteins of phages infecting the same host are suggested to employ similar strategies. All of them appear to share a tendency to interact with central “hub” proteins, highlighting their potential disruptive effect on the host metabolism. Another commonality is found in the targeting of proteins involved in transcription, replication, recombination, and repair functions [38].

The recent combination of Y2H with high-throughput sequencing technologies (HTS) overcomes labor-intensive clone-by-clone analysis and has been shown to speed up the study of PPIs while increasing the efficiency and sensitivity of the method. Different approaches were implemented, including recombination-based methods [43,44,45] and methods based on genomic library screening against one single protein [46,47]. Although these techniques represent a marked improvement of the method, the data analysis and interpretation remain a challenge. Indeed, the large amounts of data generated with these approaches require the development of specific bioinformatics pipelines, and fine-tuning of thresholds to select reliable interactions.

To date, few large-scale analyses of phage–bacteria PPIs have been conducted. These works focus on model viruses from the order *Caudovirales* [35,36,37,38,39] and, to our knowledge, no detailed studies on phage-host PPIs have been performed on other groups of phages, including the family *Tectiviridae*. Besides, these existing studies rely on Y2H screens of individual clones. In our work, we used a novel yeast two-hybrid–high-throughput sequencing approach (Y2H-HTS), aiming to obtain a proteome-wide virus–host protein interactome between the betatectivirus Bam35 and its host, namely, *B. thuringiensis*. By performing a total of 156 Y2H assays, we established a highly selective interactome Bam35–*B. thuringiensis* in which we could detect patterns within the phage–host interactions and identify specific interactions to better understand viral protein functions and phage biology.

## 2. Results

### 2.1. Integrating the Yeast Two-Hybrid System with high-throughput Sequencing for High-Confidence Interaction Datasets

To obtain an extensive protein–protein interactome of Bam35 and *B. thuringiensis* by developing a novel and customizable approach, we established an experimental setup that combined traditional yeast two-hybrid with high-throughput sequencing methods (hereafter Y2H-HTS). We used the previously generated Bam35 ORF collection [19], containing bait constructs for all 32 ORFs of Bam35, which were cloned in both orientations (C- and N-terminal fusions to the Gal4p DNA binding domain (DBD)). This collection also includes truncated versions of proteins (labeled with a “t”) with a predicted transmembrane domain, from which this domain was removed. The Bam35 ORF constructs were used to screen two newly generated genomic libraries of *B. thuringiensis*. 

**Figure 1:**
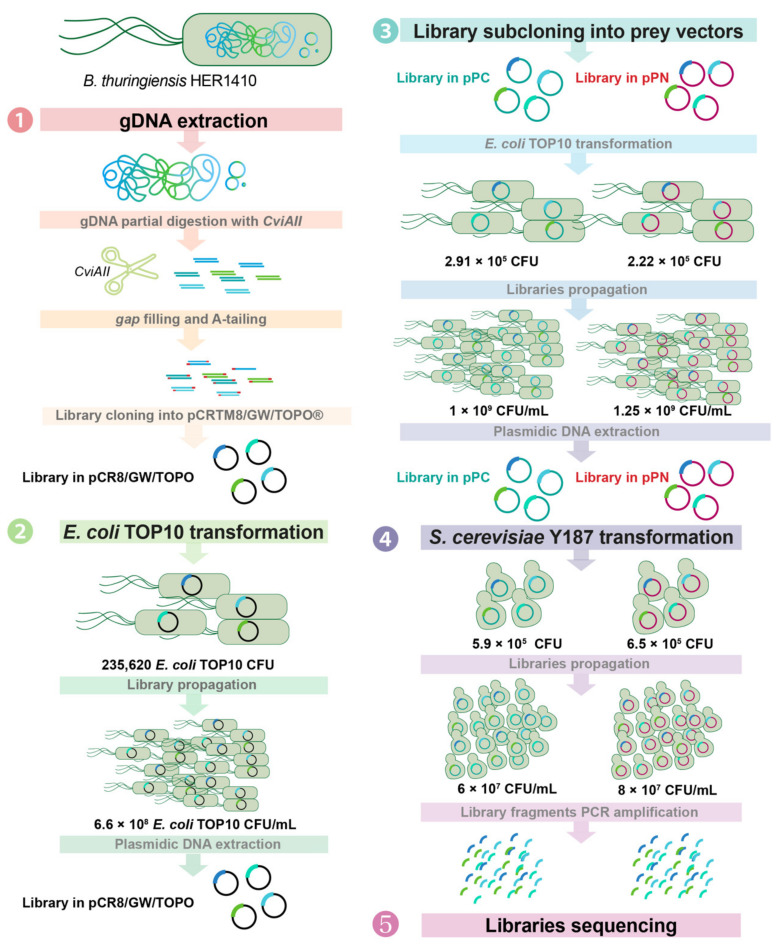
Y2H genomic library construction workflow. (**1**) The genomic DNA of *B. thuringiensis* HER1410 (blue-green) was partially digested using *CviAII* and cloned into the pCRTM8/GW/TOPO vector (black circles), generating the HER1410 genomic library in pCRTM8/GW/TOPO. (**2**) This library was used to transform *Escherichia coli* TOP10 cells that were propagated and used for plasmid extraction. (**3**) The library in pCRTM8/GW/TOPO was subcloned into the Y2H prey vectors pPC (teal circles, DNA-binding Gal4p activation domain (AD) fused at the C-terminus of the genomic fragment) and pPN (red circles, AD fused at the N-terminus), generating genomic libraries in pPC and pPN. After the transformation, propagation, and plasmid DNA extraction, both libraries were used to transform *S. cerevisiae* Y187 (**4**). Libraries in *S. cerevisiae* were propagated, plasmid-extracted, PCR-amplified, and sequenced using Illumina HTS (**5**, see below and Figure 2). The number of independent clones for each transformation step is indicated.

The genomic libraries of the *Bacillus thuringiensis* HER1410 genome (6,147,475 bp) that is suitable for Y2H were obtained using a four-step procedure (Figure 1). These libraries were generated via the partial digestion of the genomic DNA followed by insertion of the obtained fragments into the Y2H prey expression vectors pGADCg and pGADT7g (pPC and pPN), respectively generating C-terminal and N-terminal fusions of DNA-binding Gal4p activation domain (AD) to the Gateway cassette.

**Figure 2:**
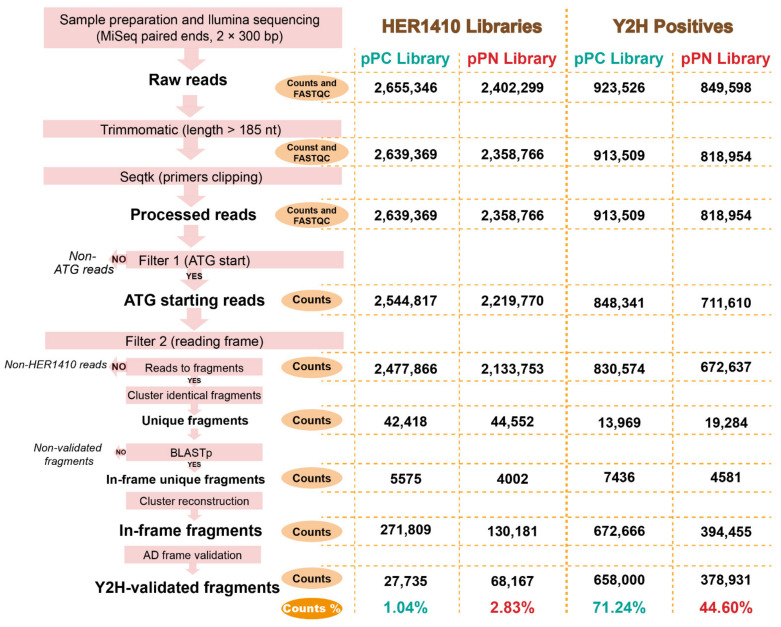
Analysis of the high-throughput sequencing data from the *B. thuringiensis* HER1410 genomic libraries and Y2H positive hits. Bioinformatics pipeline for the Y2H-HTS data analysis. Schematic representation of the different steps that were followed for sequencing the data processing of Y2H libraries and Y2H positives. After each step, the number of reads or fragments was calculated (counts). For each library, these counts are indicated in the two columns on the right (pPC: teal, pPN: red). For the Y2H positives, only the reads over 300 bp are detailed, see Supplementary Figure S9 for the complete analysis. The final percentage of validated reads is indicated. In the first few steps, FastQC was used to evaluate the quality of the reads, as illustrated in Supplementary Figure S3.

Both libraries were analyzed in detail using Illumina sequencing. A custom bioinformatics pipeline (Figure 2) was generated to process the HTS data of the HER1410 libraries, as well as the yeast two-hybrid positive hits (see below). Regarding the Y2H libraries, selected fragments of the libraries containing ATG-starting reads (97% for pPC and 96% for pPN) were converted into HER1410 fragments that, after clustering, yielded at least 40,000 different HER1410 fragments for each library. Mapping of these unique fragments against the host genome resulted in a coverage of 40% of the total nucleotides of HER1410, which was almost identical for both libraries (40.8% for pPC and 40.07% for pPN). Furthermore, both libraries were equally distributed throughout the genome with similar coverage (Supplementary Figure S2). Two additional filters were applied to the unique fragments based on their translation and position relative to the *GAL4-AD* gene (the GAL4p activation domain-encoding part). The final Y2H-validated fragments represented a total of 417 genes of HER1410 (7.2%) in the pPC and 781 genes (13.5%) in the pPN library (Supplementary Table S1). Although both libraries initially contained a similar distribution of ATG fragments, the difference in the number of validated genes represented by each library, which was lower for pPC, was mainly due to the differential final filtering step, which was more stringent for this combination (see the Materials and Methods section). 

**Figure 3:**
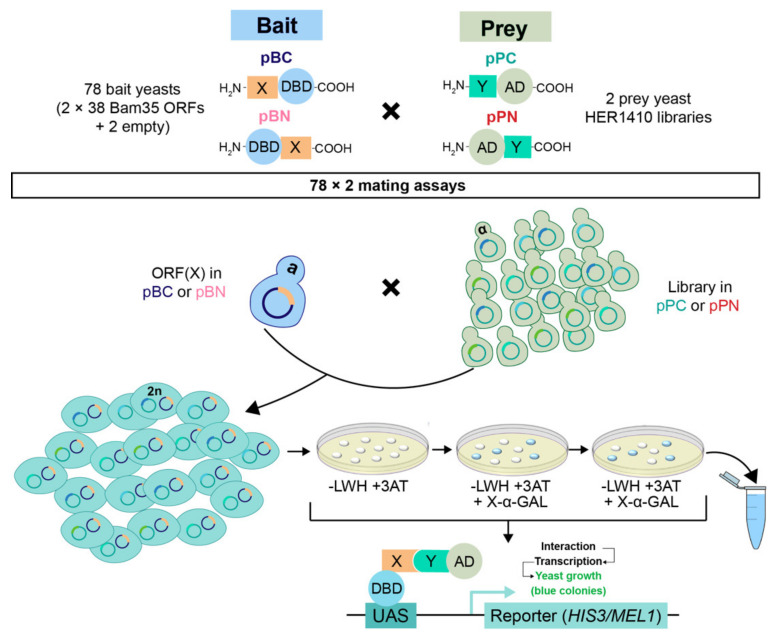
Bam35–*B. thuringiensis* Y2H interactome screening. Schematic representation of the Y2H assays. A total of 156 Y2H experiments were performed to test the interactions between each of the ORFs of Bam35 (X) that were cloned into bait vectors (C- and N-terminal fusion) and the generated HER1410 libraries (Y) that were cloned into prey vectors (C- and N-terminal fusion). For each assay, a Bam35 ORF-containing bait yeast was mated with one of the HER1410 library-containing prey yeast. Positive interactions were detected using *HIS3* and *MEL1* markers. Mated cells were plated on selective solid media without leucine, tryptophan, or histidine (-LWH); supplemented with the corresponding 3AT concentration; and then replicated twice in -LWH +3AT plates supplemented with X-α-Gal. The cells that were able to express the *HIS3* gene grew on -LWH plates and, further, those that were able to express *MEL1* turned blue in presence of X-α-Gal. All cells of the last replica were pooled and harvested for further analysis.

The interaction analysis of all viral ORFs against the genomic library in both orientations resulted in a total of 158 different possible combinations (Figure 3), including control mating experiments of our libraries with the empty bait vectors. However, given that the ORF16 of Bam35 was not available in the pPC vector [19], this number was reduced to 156 individual Y2H assays. For each assay, a bait yeast harboring the Bam35 ORF(X) bait expression vector was mated with one of the prey libraries. The overall calculated mating efficiency was 61.23%.

Positive interactions were first detected using the *HIS3* reporter gene by plating the mated cells on selective media lacking histidine (Figure 3 and Supplementary Figure S4A). To increase the stringency of selection when using the *HIS3* reporter gene, autoactivation of the bait proteins was minimized by adding the optimal concentration of the inhibitor 3-amino-1,2,4-triazole (3AT) for each bait construct. Furthermore, the reduction in cell background and, therefore, the efficient growth and selection of positive colonies was achieved via two steps of replica plating (Supplementary Figure S4A). As can be observed in Supplementary Figure S4A, the screening led to a wide variety of results in terms of color and number of colonies. The assays that included the pPN library were plated on selective media with low 3AT concentrations (0–0.1 mM) resulted in a pink lawn (Supplementary Figure S4A, lane 4). This could correspond to a false positive background caused by the pPN library, which prevented the recovery of positive colonies. For these cases, the increase in 3AT concentration up to 3 mM effectively prevented the pink lawn, enabling the growth of previously hidden interacting partners (lane 5). In addition, based on the *MEL1* reporter selection system, 5-bromo-4-chloro-3-indolyl α-D-galactopyranoside (X-α-Gal) was added to identify and amplify reliable interactions by increasing the 3AT concentration in the plates containing mostly white colonies (weak interaction). However, the plates meeting this requirement already contained the highest concentration of 3AT. To identify the prey interaction partners in each assay, cells from the last replica plate (Y2H positive clones) were pool-harvested and the prey fragments were amplified using PCR for subsequent Illumina sequencing (Supplementary Figure S4B). The PCR products of each assay showed distinct patterns of discrete bands or a smear, which corresponded to different sizes within the library size range. Nine combinations showed few or no colonies and, consequently, no PCR product was generated (Supplementary Table S2).

High-throughput analysis of each Y2H assay using Illumina sequencing (300 bp paired-end run) resulted in 12,229 reads per sample on average (Supplementary Table S2). These reads were filtered using the bioinformatics pipeline illustrated in Figure 2. After the data treatment, mapping of the Y2H-validated fragments allowed us to retrieve a total of 4477 possible interactions (Table 1, Supplementary Table S3). A detailed analysis of these results, as described in the Supplementary Material, revealed that the Y2H screening strongly favored fragments in the frame with the *GAL4-AD* gene and the actual ORFs within the genome. Only a minor library-borne background was observed, primarily for combinations that included the pPN library. The 4477 interactions were classified according to their enrichment or presence in the Illumina reads dataset as follows: A (100–10% of normalized counts), B (10–0.25%), and C (0.25–0%). These categories were validated via a small-scale sequencing analysis, which showed a 100% recovery of prey fragments by HTS (except for IS*4* transposases) and a good correlation between their presence in the sample and their enrichment category (see the Supplementary Material for details).

A similar number of total interactions, around 1000, were found for each of the bait–prey combinations CC, CN, and NN (pBC–pPC, pBC–pPN, and pBN–pPN, respectively), although their distribution into categories varied. Indeed, the major contributor to the category A interactions was the CC combination, while the NN combination generated the lowest number of A interactions. On the other hand, the NC (pBN–pPC) combination resulted in at least one third more interactions than the other combinations, contributing more to B and C total interactions, but similarly to category A. Overall, compared with the pPC library interactions, those involving the pPN library showed a higher percentage of reads in category C. 

Remarkably, although a total of 3334 interactions out of the 4477 were grouped in category C, they corresponded to only 2.04% of the total reads (Table 1). These hits could not be properly discriminated from the data noise (see the Supplementary Material) and were excluded from the main results to increase the specificity, resulting in a dataset of 1143 interactions (Table 2). This is consistent with Y2H methods sometimes having a high rate of false positives [48]. In addition to the use of the appropriate 3AT concentration to prevent autoactivation by baits, two data filters were used to remove false positives. First, potential “sticky” preys, i.e., promiscuous prey fragments that interact with more than the average number of different bait interactors (six), were identified. A total of 36 “sticky” prey (Supplementary Table S4) were removed, reducing the dataset to a total of 228 filtered interactions (Table 2). Second, the prey CDS fragments that were identified in diploid yeast harboring the empty plasmid (Supplementary Table S5) were also tagged as false positives and deleted from the dataset. Due to their tag as “sticky” prey, most of the prey fragments detected in the empty combinations had already been removed with the previous filter. Therefore, this second filtering step resulted in a reduction of only 17 hits. 

Lastly, after the consolidation of the duplicated results, a final high-quality dataset of 182 PPIs was obtained (Supplementary Table S6). More than half of these interactions (106) were found with the NC bait–prey pair. The second pair retrieving more filter-passing interactions was the NN pair, which suggested a higher degree of detection of putative interactions for the N bait fusion. A total of 13 interactions were detected in two different bait–prey pairs, and, interestingly, seven of them involved the Bam35 membrane structural component P25 (Supplementary Table S6).

### 2.2. Challenging Y2H Single Hits from the Fragment Genomic Library Using ORF Pairwise Y2H Assays

To further validate the detected interactions from the Y2H-HTS screening, which were obtained using computational analysis of the original Illumina dataset, 33 randomly chosen interactions were re-evaluated (Table 3). In this case, binary Y2H was used to individually test the interactions between the complete host proteins and their putative viral partners. Prey vectors containing the selected HER1410 ORFs were obtained and assayed with the correspondent bait vectors, resulting in seven confirmed interactions (Supplementary Figure S5). On the other hand, twelve interaction pairs could not be confirmed since even though the yeast expressing both Bam35 and *B. thuringiensis* proteins was able to grow, the maximum 3AT tolerated was equal to or smaller than that of the yeast expressing only the prey or bait protein (Table 3). Strikingly, only NC interactions could be confirmed using this method, coinciding with the bait–prey combination that is more commonly found within the HTS-predicted interactions. Most of the confirmed interactions were detected at high 3AT concentrations, which indicated a strong interaction. 

In this case, rather than the enrichment category, the percentage of the protein sequence covered by the original library fragment was found to be a relevant indicator for the full-length-fragment correlation confirmation rate. Thus, all the interactions showing a host protein coverage above 74% were validated, while the rest (coverage under 54%) could not be validated for the full-length protein (Table 3). On the other hand, although only 21% of the retested interactions could be confirmed with this method, we cannot rule out that true interactions between viral proteins and host protein fragments or domains, which could have been hidden within the full-length construct, occurred. In conclusion, the confirmed positives rate provided a high confidence level for the interactions detected in our screening, at least for those involving high coverage fragments. These results suggested that the protein coverage of these fragments could also be useful as an additional confidence score in the Y2H-HTS approach to link protein fragment interactions with full-length PPIs.

### 2.3. Bam35–B. thuringiensis Y2H-HTS Predicted the Interactome: Clear the Forest to Predict PPIs 

**Figure 4:**
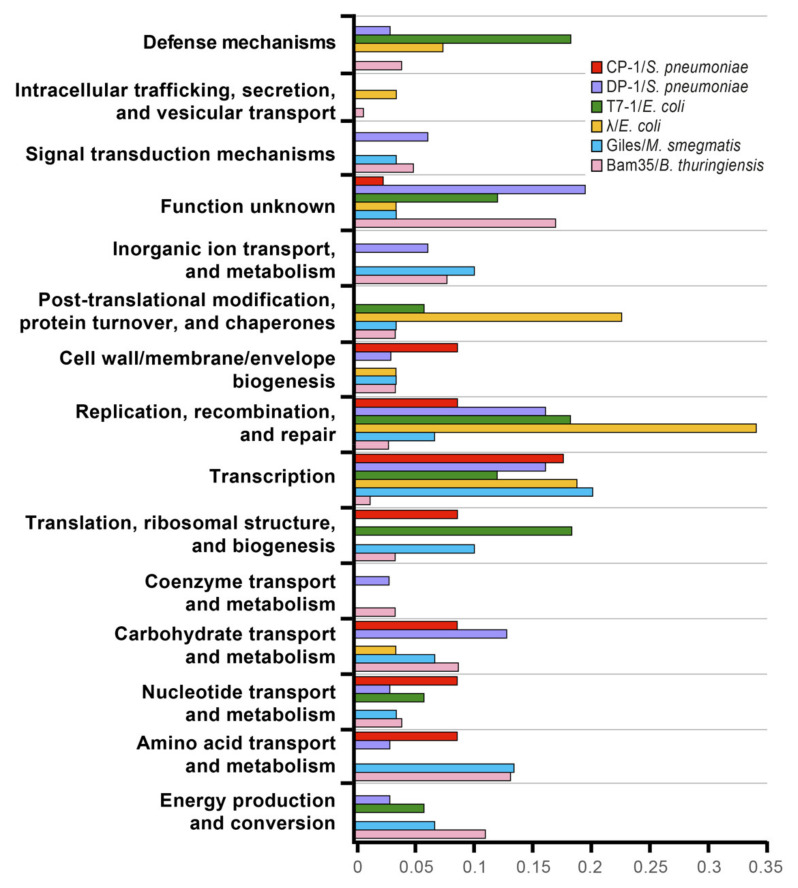
Interactions between functional groups of proteins. Stacked bar chart representing the proportion of interactions between the functional groups of Bam35 and the COG groups of *B. thuringiensis*. The X-axis represents the percentage of interactions that involved a defined COG group out of the total interactions that involved a defined Bam35 functional group. The number of total interactions is indicated for each combination inside the bars.

From the final dataset including the 182 selected interactions, 54% included viral proteins that were functionally linked to the “Virion structure and DNA packaging” functional group (Figure 4). Among them, both P26 and its non-transmembrane domain variant (P26t) retrieved the highest number of interactions (17 and 15 respectively), followed by P25 with 11 interactions (Table 4). 

In general, host metabolic proteins seemed to interact to a higher extent with viral proteins than the rest of the clusters of orthologous groups (COGs), with more than half of the total PPIs including this type of proteins (Figure 4). Interestingly, viral proteins involved in “gene regulation and genome replication” targeted “information storage and processing” host proteins in a higher proportion compared to other viral groups, indicating a possible link between these two functional groups. As shown in Supplementary Figure S6, these host partners did not connect different viral functional groups but remained restricted to the replication and regulation of viral nodes. The different viral functional groups of proteins seemed to be connected mostly by metabolism host proteins. However, no connection was observed between “host recognition and cell lysis” and “gene regulation and genome replication” groups, and only one node linked the three groups of viral proteins. This interactor could indeed be a hub due to its role as an aminopeptidase, which is a cytosolic protein that is presumably involved in the processing and regular turnover of intracellular proteins [49]. 

**Figure 5:**
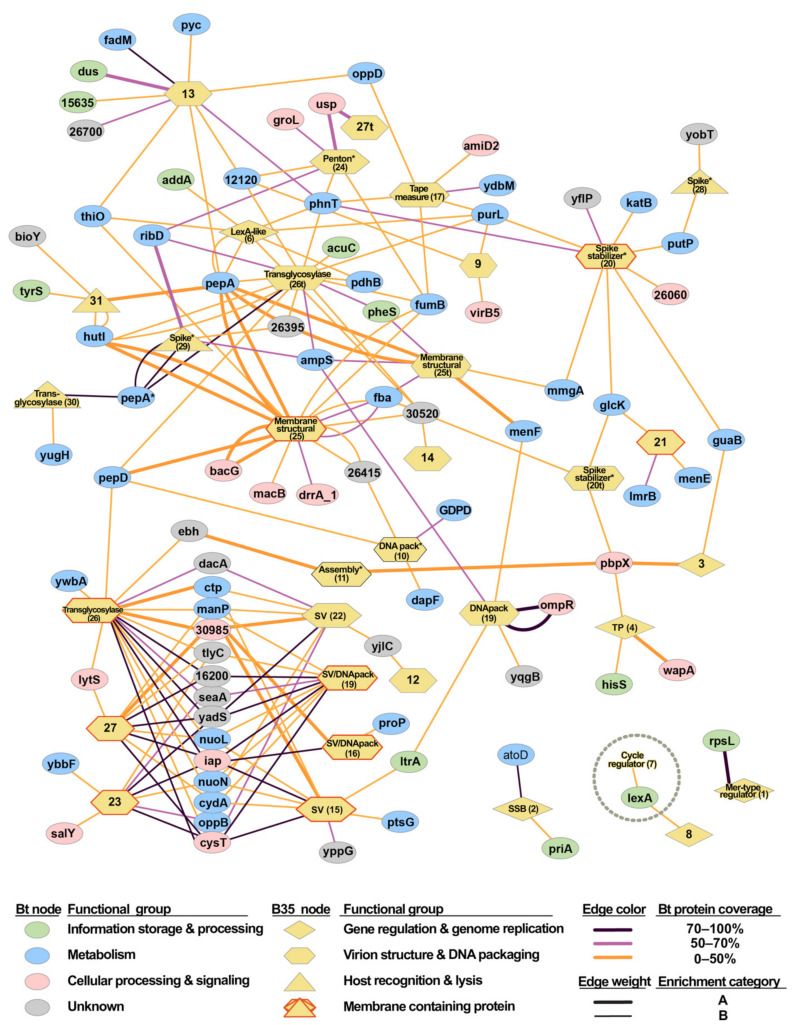
Bam35–*B. thuringiensis* protein interaction network based on the Y2H–Illumina screening. Network of the 182 detected interactions (195 bait–prey pair combinations) between the host proteins and the viral proteins (Supplementary Table S6). Host proteins (Bt nodes) are represented by ellipses colored according to their functional groups and the viral proteins are represented by yellow nodes shaped according to their functional groups. Viral nodes are labeled by their known or suggested (*) function and their gene number (between brackets) is indicated in Supplementary Figure S1. SV stands for special vertex localization. Host proteins are identified by their annotated function or, for the unknown function proteins, their locus number in the HER1410 genome (accession numbers available in Supplementary Table S6). Each interaction (bait–prey combination) is represented as a line connecting two nodes. Line colors indicate the coverage of the host protein by the detected prey fragment, where the line weight indicates the enrichment category of the interaction (B for prey proteins detected as 0.25–10% of the sample counts and A for 10–100%). The interaction between the viral P7 and the host LexA that was detected in this work and previously published in Caveney et al. [28] is indicated with a gray circle. PepA* refers to the M42 family metallopeptidase that is encoded by the host gene HBA75_23575 and PepA refers to the cytosol aminopeptidase that is encoded by the host gene HBA75_24805.

Analysis of the Bam35–*B.thuringiensis* interactome also allowed for the identification of specific patterns and remarkable interactions (Figure 5 and Supplementary Table S6). Importantly, the only previously characterized Bam35–*B. thuringiensis* PPI, which consisted of the interaction between the viral P7 and the host LexA protein [28], was detected in our final dataset. Interestingly, according to our results, this host protein could also interact with the viral protein P8, which is a protein of unknown function that, similar to P7, belongs to the “gene regulation and genome replication” functional group. Moreover, as explained above, the non-transmembrane variant of the protein P26 (P26t) appeared to be a hub in the interactome since several host interactors were identified that also interacted with other viral proteins. Furthermore, the viral membrane protein P25 appeared to be a structural hub that partnered with several transport proteins. On the other hand, in agreement with the intraviral interactome results [19], the complete protein P26 interacted with host proteins that were also linked to several structural proteins, especially those that are involved in the special vertex formation (P15, P16, P19, P22), as well as P23 and P27, which are structural proteins of unknown function. Therefore, these proteins appeared to form a cluster that was connected by a high number of host proteins, many of them linked to membrane-related transporters (Supplementary Table S6). Importantly, this “special vertex cluster” contained several high-confidence interactions, including validated interactions, category A interactions, and fragments that significantly covered the complete host proteins. 

Contrary to structural viral proteins that are highly interconnected with host proteins, those involved in “gene regulation and genome replication” interacted independently. This was the case, for example, for the B35SSB (P2) [50], which remained outside of the network, interacting only with an acetate CoA transferase and a primosomal protein. In general, the obtained network showed a highly interconnected system, where some viral proteins could act as hubs in the bacteria–virus interactome [19]. Lastly, no interactions above the thresholds were detected between the host proteins and the viral P5 (DNA polymerase); P18 (major capsid protein), which was somewhat expected by their function; and P32. P32 overlapped with half of the P30 sequence (see Supplementary Figure S1) and, accordingly, both proteins shared most of the interactions in the intraviral interactome [19]. However, in this work, the two interactions of P30 were not detected with P32, even in the raw data, indicating that those interactions were mediated by the N-terminal half of P30.

## 3. Discussion 

### 3.1. A Proficient Y2H-HTS Method to Detect Multiple Phage–Host PPIs

To date, few attempts have been made to obtain bacteria–phage interactomes. Among them, the most complete includes an ORF collection of 3974 (94%) of the known *E. coli* K-12 CDS and 68 out of 73 CDS of phage lambda, whose interactions were tested using a pool-arrayed screening approach [36,51]. This method was also used in the case of *Streptococcus pneumoniae* and its phages, although with a less representative host ORFeome [38]. Others turned to fragment-based approaches for PPI analysis, like those of *Pseudomonas* phage ϕKMV and that of mycobacteriophage Giles [35,39]. However, those approaches require ORFeome libraries and/or individual clone identification, which negatively impacts the efficiency and the potential generalization of the method to less characterized models. These limitations prompted us to use a combination of a random fragment library, and a novel pipeline, which allowed us to benefit from high-throughput sequencing to study the interactome of Bam35 and its host, namely, *B. thuringiensis,* and which could readily be implemented toward other systems. Coupling Y2H and HTS can generally be performed using two strategies. An all-versus-all strategy using library recombination approaches or the use of barcode indexing, which enables simultaneous sequencing of interacting preys from multiple separate assays in a single Illumina paired-end run [44,45,47,52]. In our case, the latter proved more appropriate since Bam35 has a limited number of viral genes that were already available and tested for autoactivation in Y2H [19]. We also generated and thoroughly analyzed the random fragments genomic library of *B. thuringiensis* HER1410, which is a strain that is highly sensitive to *Bacillus* phage infections [17,53]. Based on this, our custom HER1410 library and the 32 annotated CDS from phage Bam35 were screened using Y2H. This resulted in a large and high-throughput screening comprising 156 assays (see Figure 3), which potentially tested more than 80,000 protein–protein pairs. A total of 4477 interactions were initially detected, which were filtered and ranked to establish a threshold that differentiated low confidence interactions (or the background) from reliable interactions. In our work, we showed that the Y2H-HTS approach was highly selective for in-frame fragments, whose presence in the original library was at 1–2%, in line with previous work [54]. Indeed, some prey fragments from the Y2H positive results were not found in the libraries’ sequencing data. This suggested that these prey were scarce within the libraries and could not be detected, despite the high sequencing depth of the library (over 200×). Altogether, our results indicated that a higher depth could reveal the presence of additional fragments, increasing the calculated quality of the library. 

The establishment of categories based on the enrichment of the prey fragments per sample was shown to keep one-fourth of the interactions while retaining 98% of the Illumina reads (Table 1). Moreover, as previously proposed [46,47,55], the enrichment of the interactions, i.e., the normalized number of sequencing reads, could reflect higher affinity between the pray and bait, as more stable interactions would be more common among the pooled positive colonies. In line with this, the validation of these categories by single-colony sequencing confirmed the strong correlation between the number of positive colonies and their enrichment in the Illumina data. We could therefore conclude that the analysis of the results provided a quantitative value to the dataset, with reduced bias and high sensitivity. 

It is widely recognized that one of the main limitations of Y2H is the high rates of false positives and false negatives. Caufield et al. [56] showed that the false-negative rate can be considerably reduced through the use of different vector pairs. Particularly, permutations of C- and N-terminal Y2H vectors increase the coverage of interactome studies, reduce the number of false negatives, and detect strong interactions [57]. However, for bacterial and phage–bacteria interactomes that include ORF or genomic libraries, these libraries are usually cloned in the N-fused variant for both the prey and bait, possibly due to the restrictions imposed by the difficulty of creating “in-frame” C-terminal fusions [35,38,39,58,59]. In this work, the C-terminal fusion library gave rise to a high number of putative interactions when combined with the N-fusion viral proteins, despite harboring fewer in-frame fragments. A total of 13 out of 182 putative interactions were found in more than one combination, showing a lower overlap than for intraviral interactomes [19].

We also used several methods that aimed to maximally reduce false-positive interactions, such as the competitive inhibitor 3AT, which prevents bait self-activation, the addition of negative controls including the empty plasmids, and the identification and removal of “sticky” prey. Remarkably, a large percentage (97%) of the prey proteins that were found in the empty combinations were also tagged as “sticky.” Importantly, the high-throughput technique that was used in this work allowed for the identification of all prey partners, enabling the effective discrimination of specific interactors from “sticky” proteins and other false positives, as suggested elsewhere [47,60]. After the efficient removal of a high number of false positives (85%) by these filters, 182 candidate interactions could be identified. 

Validation of high-throughput study results is hardly feasible, though confidence scores have proven useful [47,61,62]. In addition, Y2H approaches can validate their methods by retesting the interactions with pairwise Y2H involving the constructs that were detected in the first screen [36,47]. In our case, we implemented an alternative validation approach in which we investigated the correlation between interactions involving complete proteins and their domains, essentially challenging the screen. Interestingly, only interactions whose initial prey fragment highly covered the complete protein (>74%) were positive in the new assay. However, it should be noted that the unconfirmed interactions from this method should not be excluded from biologically relevant interactions. Indeed, although some interactions that require full-length proteins could be missed [52], the use of protein fragments can increase the sensitivity of the screening, as shown in Yang et al. [45], where some known interactions were only detected when domains or domains vs. full-length proteins were assayed. Likewise, the previously described interaction between LexA and P7 [28] was detected in our screen with a fragment covering 42% of the protein. Therefore, notwithstanding the limitations of the fragment-based Y2H, our results indicate that the use of a random fragment library not only expanded the interaction space but could also be useful for domain interaction determination and additionally provided a protein length coverage cutoff criteria.

### 3.2. The Bam35–Bt Y2H Interactome Revealed the Clustering of Special Vertex Proteins and a Wide Modulation of Host Cell Metabolism

As plasmidial prophages, betatectiviruses have a particular lysogenic cycle that raises many questions about their evolution and maintenance [63,64]. Disclosing the virus–host interactions at the molecular level would shed some light on this growing group of enigmatic viruses and their evolutionary synergy with relevant human and animal pathogens. Based upon enrichment, specificity, and biological meaning, we could obtain a virus–host interactome that certainly allowed us to identify some common patterns and prey partners of interest.

**Figure 6:**
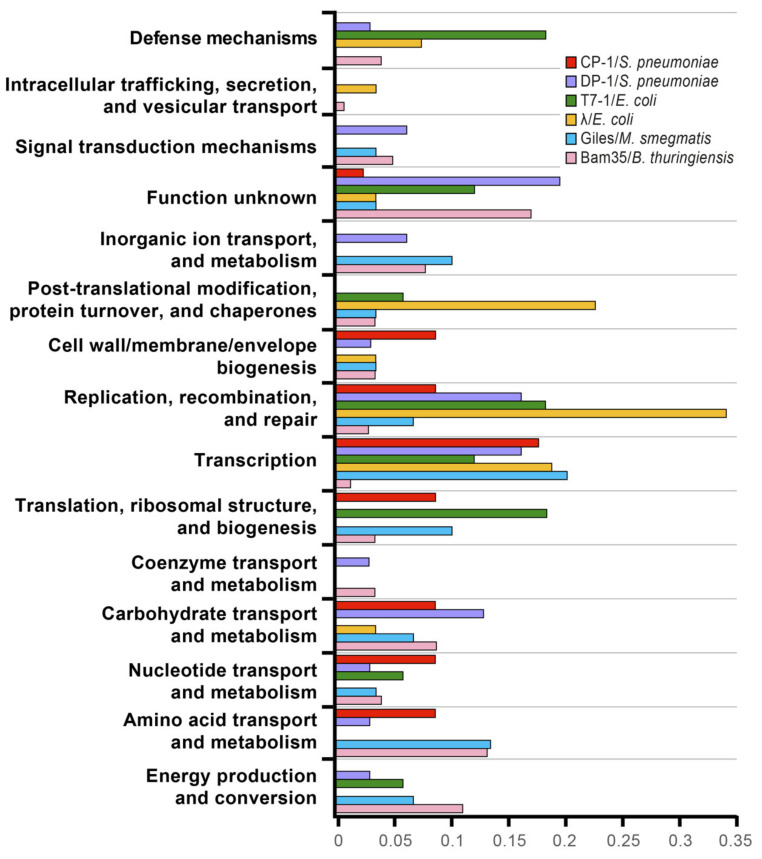
Frequency of phage-targeted proteins and their functional classes adapted from Mariano et al. [38]. The interactions between Giles and *Mycobacterium smegmatis* (blue) [39] and the interactions detected in this work between Bam35 and *B. thuringiensis* (pink) were added.

In previous works, structural proteins were usually not involved in host–virus interactions as they are mainly involved in most intraviral interactions [36]. Conversely, a high proportion of the detected Bam35–*B. thuringiensis* PPIs are involved in “virion structure and DNA packaging.” When compared with previous works on phage–host interactomes, functional annotation frequencies of the interacting host partners do not share similar patterns (Figure 6). Strikingly, although a higher frequency in protein processing and gene regulation was associated with phage lambda’s lysogenic state, these categories were not specifically represented for Bam35 and Giles phages, which are also temperate. Nevertheless, we did detect protease enrichment (see below), which is known to be important for phage lambda, among the Bam35–host interactions. Previous phage–host research mainly identified host proteins that are involved in transcription, replication, recombination, and repair functions. However, the Bam35 proteins involved in DNA replication showed very few interactions. Furthermore, in agreement with an episomal lysogenic stage in which the viral genome could be replicated only by viral factors, host proteins involved in DNA replication and recombination were uncommon targets. In turn, phage proteins largely targeted host metabolic processes and transport proteins, which would be modulated or hijacked during viral lysogeny and/or lytic cycle development. Among them, host partners involved in menaquinone metabolism (MenF, MenE) should be highlighted here. Menaquinone (Vitamin K12) is involved in anaerobic metabolism and is essential for complex colony formation [65], which is a process that is strongly influenced by betatectivirus [29]. Similarly, two viral proteins, namely, the spike stabilizer P20 and the protein of unknown function P21, interact with the host glucokinase (GlcK), which might be related to a previously reported faster glycogen metabolism in the GIL01 and GIL16 lysogenic strains [29].

Interestingly, despite the high differences in both the virus and the host, the Giles–host interactome approach is the most similar to ours since it is based on a host genome fragments library; Giles is also a temperate phage, and the results also pointed to a high viral influence in the host metabolism [39]. Furthermore, in line with our results but using a different approach, a high-throughput proteomic analysis of the effect of the betatectivirus-related plasmid pBClin15 on its host revealed a significant impact of pBClin15 on different pathways of central metabolism during growth [66]. Similarly, diverse RNA-seq analyses highlighted the influence of phages in membrane proteins, transporters, and metabolism [67,68,69,70]. On the other hand, some host interactors that were detected in our study were similar to those found in other phage–host interactomes, including ABC transporters, NADH-quinone oxidoreductases, primosomal proteins, and phosphate regulatory proteins [36,38,39].

Among the 182 different interactions, 12.6% involved different types of peptidases. PepA (locus HBA_24805) is linked to the three functional groups of viral proteins (Supplementary Figure S6). This protein presumably functions in the processing and regular turnover of intracellular proteins, which might be subverted during the viral infection. This could also be the case for a putative M42 metalloprotease (locus HBA75_23575, also annotated as PepA) and the aminopeptidase AmpS, which interact with Bam35 proteins from the structural module, including the lytic proteins P26 and P30. Accordingly, *E. coli* gene expression analysis during bacteriophage PRD1 infection showed that many proteases were highly induced during virion assembly [67]. Interestingly, the orthologs of these three peptidases in *Bacillus subtilis* interact with each other (String database) [71]. In line with this, proteases are involved in lambda–*E. coli* interactions and linked to the viral cycle regulation, although these interactions could not be detected using Y2H, probably due to their weak and transient nature [36,40]. Another protease that was found in the interactome, namely, PepD, was reported to negatively affect biofilm formation [72]. Therefore, the detection of PepD as a host target, as well as a spore coat protein, could also be linked to the influence of Bam35/GIL01 lysogeny on the *B. thuringiensis* sporulation rate and biofilm formation [29].

The Bam35 inner membrane is generated by recruitment of the host lipid membrane and results in a modification of membrane thickness and curvature. This process was proposed to be carried out by a cluster of viral proteins that includes the phage tape measure (P17) and major capsid proteins (P18) [12]. Interestingly, P17 interacted with P18 but also with transmembrane-containing proteins P25 and P26, which, in turn, interacted with several host proteins, including transporters that were associated with the host membrane (Figure 5). P25 is the second most abundant protein in purified Bam35c particles [11] and thus could play a key role in the host membrane modifications. The P26 variant without the transmembrane domain also lacks a C-terminal region of unknown function, which may explain the different sets of interaction between P26 and P26t (Figure 5), as previously reported [19]. Moreover, the lack of P26t in the special vertex cluster suggested a role of the removed 177–250 residues in these interactions. On the other hand, the P26t variant shared prey partners with P17, P24, and P29, which are also direct interactors. These results are in line with the putative role of P26 as a scaffold protein for other structural elements from the inner membrane and the viral capsid [19], as well as with its proposed function as a hub for the viral–host interaction.

Several viral proteins were clustered in the Bam35–host interactome by their common interactions with numerous bacterial nodes (Supplementary Figure S7). The suggested function for four of them (P15, P16, P19, and P22) was associated with the special vertex [11,19]. The clustering of these proteins in a similar host–phage interaction environment supported their proposed function. On the other hand, the localization of PRD1 P15, which is the Bam35 lytic enzyme P26 counterpart, in the special vertex is controversial [73,74]. Interestingly, in Bam35, our phage–host interactome suggested that P26, along with the proteins of unknown function P23 and P27, would be part of the special vertex cluster (Supplementary Figure S7). P23 has a transmembrane domain and almost all of its interaction partners include different transporter proteins, hinting toward a role as a membrane-associated protein that is related to the special vertex. P27 is also a transmembrane protein and was suggested as the penton protein [12], although the intraviral interactome results downplayed this possibility [19]. The non-transmembrane variant of P27 only interacts with a host N-acetylmuramoyl-L-alanine amidase, which is a protein that degrades the cell wall peptidoglycan. This host protein also interacts with P24, which is proposed to be the penton protein and is related to the transglycosylase (P30), presumably making it responsible for the viral entry mechanism that is associated with the spikes [19,21]. Thus, P27 may be an anchoring virion protein whose function is related to both viral entry and release. These results could also suggest the recruitment in the virion of an additional host amidase that would help in the viral entry. Another interesting putative partner of P24 is a GroEL chaperonin, which is thought to assist the insertion of PRD1 proteins in the virion membrane and is essential for PRD1 assembly [75]. *E. coli*–PRD1 RNAseq data showed high expression levels of the host GroEL during assembly, while in the case of pBClin15b, which displays cryptic prophage behavior, *GroEL* was downregulated [66,67]. Thus, it is tempting to speculate that this chaperone may also have a role in the assembly of Bam35. 

Importantly, all the proteins that are clustered in the “special vertex group” have a predicted transmembrane domain [11], whereas the variants lacking this domain did not appear in this group. This could explain their interaction with the same partners, which are mostly membrane-related transporters and may help the phage to attach to the host membrane. Particularly, the strong interactions with a putative phage tail tape measure protein from the putative prophage pLUSID3 (30985 in Supplementary Figure S7) could be have been due to the protein region covered by the Y2H fragment, which comprises a predicted transmembrane domain. This domain appears specific to *Bacillus* phages and therefore would suggest interactions between elements of the mobilome. However, this protein is puzzling as *Caudovirales* lack any membrane within their viral particle. Furthermore, six out of the seven confirmed interactions belonged to this cluster (Supplementary Figure S7). Remarkably, within these, the enterotoxin/SH3 domain protein (Iap in Supplementary Figure S7) was also annotated as the conserved virulent factor EntA [53,76]. SH3 domains are known to be involved in protein–protein interactions and cell wall recognition and binding [77]. Since the transglycosylase P26 does not possess any signal peptide [21], it is also possible that the enterotoxin can be recruited by the phage to reach the membrane and the cell wall during and while contributing to lysis. 

One of the most relevant interactions that was detected with Y2H-HTS in the Bam35–HER1410 model was the previously described direct interaction between the viral P7 and the host LexA, which is key for maintaining lysogeny [18,27]. Here, the LexA fragment comprised the second half of the protein (residues 121 to 206), suggesting that the interaction domain was located in this region, and it was sufficient to establish the interaction. Importantly, the detection of this previously known (and unique) interaction between Bam35 and *B. thuringiensis* provided confidence in our method. Only one other viral protein interacts with the host LexA, namely, P8. Interestingly, the ORF8 is located in a highly variable region of tectiviruses whose ORFans may alter phage regulatory functions, influencing phage and possibly also host fitness [78]. However, the lack of sequence similarity does not provide any hints about its specific function. Since the only detected host partner of P8 is LexA, it is tempting to suggest a role of P8 in viral cycle control. Particularly, as the ORF8 is located at the end of a gene cassette that is responsible for maintaining the lysogenic cycle [18,27], the P8–LexA interaction might also contribute to the fitness and regulation of the lysis–lysogeny switch. P8 interacts with the viral LexA-like activator protein (P6) and it shares with the latter and P7 the interactions with structural proteins P24 and P26. This suggests that all the regulatory proteins could be present in the viral particle, allowing their involvement in the very early infection events, as proposed previously [19]. Moreover, P6 interacts with PepA (locus HBA_24805), whose *E. coli* ortholog is involved in transcription and recombination [79], and therefore it could also be related to the Bam35 life cycle regulation. 

Another key interaction that we expected to uncover in this work was the identification of a viral protein receptor. Several host proteins in our interactome may be candidates to be the Bam35 receptor, including membrane-associated proteins, such as metabolite transporters. As such, it is tempting to speculate that the host protein PutP Na^+^/proline symporter could be the receptor, as it showed interactions with the spike protein P28 and the putative spike stabilizer (P20). However, PutP belongs to an osmotically inducible operon [80], which downplays its role as the viral receptor under normal growth conditions. Therefore, it is likely that the viral receptor is not present in our libraries. Alternatively, this and other interactions may be missed by the establishment of a threshold, as shown in Yang et al. [45], and false positives can arise for the same reason. The knowledge of literature-based interactions was proven to be key in this matter [47]. Since our approach is the first one in the tectiviruses–host interactions field, we combined the use of several quality filters with the previous biological knowledge of the Bam35–*Bacillus* model and previous virus–host studies. Therefore, the high-throughput Y2H screening of Bam35–*B. thuringiensis*, including a high number of host proteins and tested interactions, resulted in the detection of multiple potentially interesting and novel PPIs. Although Bam35–*B. thuringiensis* interactions need to be further analyzed with other PPIs analysis techniques and their biological relevance remains to be explored further, they open the door to new types of host–phage interactions and a deeper understanding of Bam35 and tectiviruses.

## 4. Materials and Methods

### 4.1. Nucleotides and DNAs

DNA oligonucleotides were purchased from IDT (Coralville, IA, USA). 

Entry donor vectors pDONR/Zeo and pCR™8/GW/TOPO™ were purchased from Invitrogen. The yeast two-hybrid system (Y2H) expression vectors pGADCg, pGADT7g, pGBKCg, and pGBGT7g [57] were available in-house.

### 4.2. Bacterial and Yeast Strains

The *Bacillus thuringiensis* HER1410 strain was originally obtained from the culture collection of the Félix d’Herelle Reference Center for Bacterial Viruses of the Université of Laval (https://www.phage.ulaval.ca) and can be retrieved with host HER number 1410 (last accessed 12 October 2021). 

*E. coli* TOP10 was used for genomic HER1410 libraries generation. The *S. cerevisiae* prey strain Y187 (MATα, ura3-52, his3-200, ade2-101, trp1-901, leu2-3, 112, gal4Δ, met, gal80Δ, URA3::GAL1_UAS-,_ GAL1_TATA-,_ lacZ) and bait strain AH109 (MATa, trp1-901, leu2-3, 112, ura3-52, his3-200, gal4Δ, gal80Δ, LYS::GAL1_UAS-_, GAL1_TATA-_, HIS3, GAL2_UAS-_, GAL2_TATA-_, ADE2, URA3::MEL1_UAS-_, MEL1_TATA-_, lacZ) were used for the Y2H screen [81].

### 4.3. Genomic Library Construction

To generate a genomic library of *B. thuringiensis* HER1410, a four-step procedure was followed, as illustrated in Figure 1.

#### 4.3.1. Generation of the Genomic Library and Cloning into the Donor Vector 

The genomic DNA (gDNA) of *B. thuringiensis* HER1410 was isolated using the DNeasy Blood and Tissue kit (Qiagen) and concentrated via ethanol precipitation [82]. The extracted gDNA was partially digested with the restriction enzyme *CviAII* (New England Biolabs) and subsequently 5′-dephosphorylated with Calf Intestinal Alkaline Phosphatase (New England Biolabs). *CviAII* cuts on a sequence motif “CATG,” which is highly frequent on bacterial genomes, with 17,370 sites in HER1410 genome, cutting on average every 356.8 base pairs (bp). The digested DNA was separated by agarose electrophoresis and an agarose block with DNA fragments spanning between about 450–750 base pairs (marker bands) was cut out, subjected to gel-extraction DNA purification with QIAquick Gel Extraction Kit (Qiagen), and purified using ethanol precipitation [82]. The DNA fragments size was chosen to meet Illumina technical requirements and according to the gene size distribution. In HER1410, 75% of the protein-coding genes are shorter than 1 kb, longer fragments might have led to an ample generation of chimeric genes. Gap filling of the 3′ overhangs and A-tailing were performed using Taq DNA polymerase. The library was subsequently cloned into the plasmid pCR^TM^8/GW/TOPO^®^ using the pCR™8/GW/TOPO™ TA Cloning Kit (ThermoFisher). 

#### 4.3.2. Amplification of the Genomic Library in pCRTM8/GW/TOPO^®^

The donor plasmid library was transformed in *E. coli* Top10 cells (CBMSO Fermentation Facility, Madrid, Spain) using electroporation. Transformants were stored at −80 °C in the presence of 10% (*v*/*v*) glycerol. The number of independent clones was determined as CFU on LB-Agar plates supplemented with 100 µg/mL spectinomycin. According to the expression given by Clarke and Carbon [83], the colony bank size that was needed to obtain a plasmid collection that covered *B. thuringiensis* HER1410 genome at least once with a probability of 0.95 and an average fragment size of 600 bp, taking into account the six possible reading frames, was 1.842 × 10^5^ CFU. This number of clones was outreached along the construction of the library (Figure 1). The original genomic library was propagated in SeaPrep^®^ (FMC) semisolid medium [84] to minimize the representational biases that can occur during the expansion of plasmid DNA libraries [85]. The total number of clones was titered again via serial dilutions and plating on selective LB Agar plates. Then, 10^7^ cells were inoculated in 50 mL LB medium that was supplemented with 100 µg/mL spectinomycin and grown overnight at 37 °C for purification of the plasmid library DNA via Miniprep (NucleoSpin Plasmid, Macherey-Nagel). The efficiency of the fragment insertion (100%) was checked using plasmid purification of 15 clones, followed by digestion with *EcoRI*-HF (Supplementary Figure S8A) and sequencing (Supplementary Figure S8C) with the forward primer GW1 (Supplementary Table S7).

#### 4.3.3. Subcloning of the Library in the Y2H Expression Vectors 

Using 50 ng of the pCR8/GW/TOPO library as donor plasmids, the inserts were subcloned into the pPC (pGADCg) and pPN (pGADT7g) plasmids using a Gateway™ LR Clonase™ II Enzyme mix (ThermoFisher, Waltham USA), according to the manufacturer’s instructions. The LR reaction product was used to transform Top10 cells, titer, and amplify, as detailed above (Figure 1). The LR reactions with pPC and pPN resulted in C-terminal and N-terminal fusions of the Gal4p activation domain (AD) to the gateway cassettes respectively. The efficiency of insertion (85% for pPC and 100% for pPN) was checked via plasmid purification of seven clones from each library, followed by PCR amplification (Supplementary Figure S8B). These fourteen clones were also verified via sequencing (Supplementary Figure S8C) using attB1 and T7_FW primers, respectively (Supplementary Table S7). The genomic libraries in the pPC and pPN prey vectors were purified using Miniprep, as detailed above.

#### 4.3.4. Transformation of *Saccharomyces cerevisiae* Y187 with the Y2H Genomic Libraries 

The pPC and pPN libraries were used to transform *Saccharomyces cerevisiae* strain Y187 via electroporation [86]. Transformants were titered to determine the number of independent clones (Figure 1) and the library was further expanded in 50 150 mm Petri dishes with selective solid media without leucine. The grown cells were harvested in a liquid selective medium containing 25% glycerol for storage at −80 °C. Prior to the mating experiments, the final libraries were titered on selective media, and their genome coverage was analyzed using high-throughput sequencing (see below). 

### 4.4. Yeast Two-Hybrid Screening

We designed a multiple yeast two-hybrid screening with a collection of 80 bait vectors, previously characterized in Berjón-Otero et al. [19], that included C-terminal (pBC (pGBKCg)) and N-terminal (pBN (pGBGT7g)) fusions of the Gal4p DNA-binding domain (DBD) to all the 32 Bam35 ORFs plus six variants without terminal transmembrane domains, as well as the empty pBC and pBN vectors. Each of the bait vectors was tested against the prey HER1410 libraries in the pPC and pPN vectors, yielding a total number of 158 mating experiments. Since it was not possible to obtain the Bam35 ORF16 pPC construct, this vector was not included in the screen [19]. Mating experiments were performed between AH109 yeast cells harboring pBC or pBN with the Bam35 ORFs (bait) and Y187 yeast cells harboring the pPC or pPN libraries, as described in Mehla et al. [87], with specific modifications for the subsequent HTS analysis (see the next section). Briefly, 4 mL of the bait and prey cultures at an OD_600_ of 0.8–1 were mixed before harvesting the cells via centrifugation. The cells were subsequently plated on rich solid media (YPDA plates) to allow for mating. After overnight incubation at RT, the cells were harvested, washed, and resuspended in 2 mL of selective liquid media lacking leucine, tryptophan, and histidine (-LWH). To perform the interaction selection, *HIS3*, coding for an imidazole glycerol phosphate dehydratase that is necessary for histidine biosynthesis, was used as a reporter gene. In short, 100 µL of the resuspended cells were plated on selective solid media (-LWH) that was supplemented with 3-amino-triazole (3AT). To avoid self-activation via DBD-fusion proteins, each screen was performed in the presence of the 3AT concentration that was determined for each bait [19]. To measure the efficiency of mating, the culture was diluted 1:10^5^ and plated on diploid selective solid media without leucine and tryptophan. Moreover, to remove the background and further confirm the positive clones, we made two stamp replicas on triple dropout media in the presence of X-α-Gal. Blue colonies show positive interactions for two markers, *HIS3* and *MEL1*. In some cases, the screen gave rise to a lawn of cells. 3AT was increased for those screens to reduce the number of false positives (Supplementary Figure S4A). Finally, Y2H-positive colonies, i.e., yeast cells that were capable of growing on the triple-dropout media, from the last replica of each Y2H screen were pooled and harvested for further HTS analysis. Additionally, to validate the HTS results (see below), we randomly picked single colonies of the final replicas before harvesting. As such, we randomly analyzed 70 colonies, including all combinations (pBC–pPC, pBC–pPN, pBN–pPC, pBN–pPN) whose prey inserts were identified using colony PCR followed by Sanger sequencing (Supplementary Table S9). 

### 4.5. Genomic Library and Y2H Positives Analyses Using High-Throughput Sequencing

#### 4.5.1. pPC and pPN Libraries Sequencing

Validation of the HER1410 genomic libraries was achieved by sequencing each of the plasmid libraries, namely, pPC and pPN, purified from the yeast strain Y187 with the Zymoprep Yeast Plasmid Miniprep Kit (Zymo Research, Irvine, USA). Plasmid isolation from yeast cells typically yields very low amounts of DNA. As such, the purified plasmid libraries were used to amplify the genomic inserts using tailed-PCR (Supplementary Figure S8D) with the forward primers attB1_HTS for the pPC library and T7_HTS for the pPN library and the reverse primer attb2_HTS (Supplementary Table S7). To ensure faithful and proportional amplification of each fragment, Q5 High-Fidelity DNA Polymerase (New England Biolabs, Ipswich, USAE) was used, and the number of amplification cycles was limited to 20. Secondary PCR amplification was subsequently performed using the HTS Unit from the “Parque Científico de Madrid” to generate Illumina sequencing libraries. The PCR products were sequenced using a single Illumina MiSeq 300 bp paired-end run in this facility. The total raw reads that were obtained from the libraries sequencing represented an average coverage of the original genome (6,147,475 bp) of 260× for the pPC library and 234.5× for the pPN library. Prior to the construction of the Illumina sequencing libraries, the fragments were analyzed using agarose electrophoresis. The Illumina sequencing libraries were analyzed using microchip electrophoresis on an Agilent 2100 Bioanalyzer using DNA 7500 Assay Kit (Agilent Technologies, Santa Clara, USA).

#### 4.5.2. Y2H Positives Preparation and Sequencing

The Y2H-positive colonies from the last replica of each Y2H assay were pooled and harvested. This resulted in 156 samples, one per mating experiment. To identify the interacting prey partners for each sample, plasmid DNA was purified via yeast miniprep in multi-well MW96 format (Zymoprep-96 Yeast Plasmid Miniprep). Then, the HER1410 fragments were low-cycle, tailed PCR-amplified using Q5 High-Fidelity DNA Polymerase, as explained above, with the specific forward primers pPC and pPN and the reverse primer attB2_HTS (Supplementary Table S7). Moreover, each sample was confirmed using PCR with specific oligonucleotides for the corresponding viral bait gene. Finally, PCR products were sent to the HTS facility to construct barcoded Illumina libraries, which were sequenced in a single Illumina MiSeq 300 bp paired-end run to obtain about 15,000 reads per sample. Fragments were analyzed using agarose electrophoresis prior to the construction of Illumina sequencing libraries. Illumina sequencing libraries were analyzed using microchip electrophoresis on an Agilent 2100 Bioanalyzer using DNA 7500 Assay Kit (Agilent Technologies). The samples containing fragments shorter than 300 bp were split (<300 bp and >300 bp) and sequenced independently to avoid sequencing bias.

#### 4.5.3. Trimming, Quality Check, and Mapping of Illumina Reads

The Illumina reads were verified for quality using FastQC v0.11.8 and Trimmomatic v0.38 was used to exclude reads shorter than 185 bp [88,89]. To remove primers and plasmid sequences from the reads, a clipping step based on sequence size was performed using seqtk [90]. The first 39 and 139 nucleotides of R1 reads of the pPC and pPN libraries, respectively, and the first 38 nucleotides of R2 reads were removed. For the seqtk clipping of the Y2H positive sequences, the first 99 and 59 nucleotides of the R1 reads from the pPC and pPN sequences, respectively, and the last 38 nucleotides of the R2 reads were removed. FastQC reports of raw and processed reads were consolidated using MultiQC v1.9 [91]. To extract the in-frame HER1410 fragments, a custom bioinformatics pipeline was set up (Figure 2). Briefly, reads that did not start with the starting codon “ATG” were removed. These “ATG” sites corresponded to the prey fragment ends that were generated by partial digestion with *CviAII*, cloned in frame with the destination vectors. Subsequently, using the R1 and R2 reads, the prey fragments were reconstructed and clustered, generating a database of unique fragments. Clustering was performed using CD-hit software at 100% sequence identity level and identical length [92]. The unique fragments were then translated and searched in the HER1410 proteome using BLASTp [93]. Fragments resulting in a BLASTp hit longer than ten amino acids were kept as “in-frame unique fragments.” To obtain the frequency for each unique fragment, clusters reconstruction was performed, resulting in the number of reads for each unique fragment. Additionally, results were manually curated to eliminate the fragments containing a stop codon or that were not in frame with the AD domain in pPC, and the hits after a stop codon in pPN, generating our Y2H-validated fragments database. The bioinformatics pipeline is available on github: https://github.com/LoGT-KULeuven/y2h_Bam35-Bt_analysis (last accessed 12 October 2021).

#### 4.5.4. Evaluation of Genomic Library Quality

Genomic library statistics were analyzed using Excel software (Microsoft). The nucleotide coverage of the total fragments was calculated using bwa-mem [94] and samtools [95], and visualized with weeSAM version 1.5 (last accessed 12 October 2021 at https://bioinformatics.cvr.ac.uk/weesam-version-1-5/). 

#### 4.5.5. Evaluation of Raw Y2H Interactions 

The mapping of positive interactions reads resulted in the identification of 4477 possible interactions (Supplementary Table S3). The filtering of these raw results was performed to improve the specificity of the putative protein interaction set by applying a set of sequential filtering steps to the data. First, the number of reads corresponding to each interaction was normalized to the total reads in the sample. This normalized number was used as a measure of enrichment of the prey fragment in the corresponding combination and, therefore, of the strength of the interaction. Thus, this value was used to establish the enrichment categories according to the distribution of the positive hits (Supplementary Figure S11). The dataset was divided into three categories: C for interactions with 0–0.25% of abundance, B for 0.25–10%, and A for 10–100%. Only the most abundant interactions (categories A and B) were considered for further analysis. Second, prey fragments that interacted with a large number of baits were tagged as potential promiscuous or “sticky prey” (Supplementary Table S4). Thus, interactions involving HER1410 protein fragments that interacted with more than six different Bam35 proteins, which was the average number of interactors after selection, were excluded from further evaluation. Third, prey interacting partners of bait pBC and pBN empty plasmids were considered as false-positive generating prey. Interactions involving these prey plasmids were also removed from the dataset (Supplementary Table S5). Last, duplicated interactions that came from the under and over 300 bp sequencing runs were consolidated. Finally, the obtained putative PPIs that resulted from the dataset filtering were analyzed and represented as interaction maps with Cytoscape software [96]. 

Descriptive and comparative analysis of the positive interactions reads dataset (Supplementary Table S2) was performed with SPSS^®^ Statistics software (IBM). Linear correlations analysis between library fragments and interacting fragments reads abundance was performed with the ggplot package for R software [97].

### 4.6. Full-Length Protein Pairwise Y2H Assays 

A total of 33 putative interactions were re-screened using complete host proteins and their viral partners (Table 3). In total, 15 interactions from category A and 18 interactions from category B were selected at random and retested with the full-length host proteins. The *B. thuringiensis* HER1410 purified genomic DNA was used to amplify the selected ORFs with PCR using Q5 High-Fidelity DNA Polymerase and ORF-specific primers (Supplementary Table S7). These primers were designed using Geneious software [98], ensuring the presence of 20 to 30 nucleotides that were complementary to the ORF of interest and removing endogenous stop codons. The *attB1* and *attB2* sequences were added at the 5′ end of the forward and reverse primers, respectively. As described in Berjón-Otero et al. [19], tail PCR products were cloned into the entry vector pDONR/Zeo (Invitrogen) and subsequently subcloned into the corresponding prey vectors pPC and pPN. All vectors that were obtained from the cloning and subcloning steps were tested using colony PCR with ORF-specific primers. The insertion of the ORFs into the entry and expression vectors was also verified using sequencing. The expression vectors were used to transform *S. cerevisiae* Y187 following the heat-shock transformation protocol described in Mehla et al. [87]. Transformants containing prey vectors were selected on a solid medium without leucine and checked with colony PCR using pPC_HTS or pPN_HTS forward primers and the corresponding ORF-specific reverse primer (Suppementary Table S9).

Pairwise screens of the selected interactions were performed, as detailed in Table 3, using the bait vectors pBC and pBN containing the Bam35 ORFs that were characterized in Berjón-Otero et al. [19] and the obtained pPC and pPN prey vectors containing the selected HER1410 ORFs. We performed mating between yeast cells containing bait vectors and cells containing prey vectors on rich solid media (YPDA plates), including the correspondent self-activation controls with the empty vectors. Diploid cells were selected on a solid medium without leucine and tryptophan. Finally, to detect protein–protein interactions, *HIS3* was used as a reporter gene. For this, the obtained diploid cells were plated on selective solid media without leucine, tryptophan, and histidine and supplemented with different 3AT concentrations (0, 0.025, 0.1, 3, 10, 25, 50, and 100 mM). When the yeast growth was higher in the Bt–B35 combination than in the self-activation controls, this interaction was considered positive.

## Figures and Tables

**Table 1:** Frequency of interactions for each enrichment category. The table shows the total number of interactions and the proportion of sequencing reads (counts) that fell into each enrichment category (A for prey proteins detected as 10–100% of the sample counts, B for 0.25–10%, and C for 0–0.25%).

Enrichment		Number of Interactions					Read Counts (%)				
		*pBC*		*pBN*		Total	*pBC*		*pBN*		Total
Category	Range	pPC Library	pPN Library	pPC Library	pPN Library		pPC Library	pPN Library	pPC Library	pPN Library	
**A**	**10–100%**	93	43	67	27	230	77.89	72.61	73.75	81.09	75.93
**B**	**0.25–10%**	262	165	391	95	913	20.76	24.34	24.52	15.76	22.03
**C**	**0–0.25%**	738	794	990	812	3334	1.35	3.05	1.73	3.15	2.04
**Total**		1093	1002	1448	934	4477	100	100	100	100	100

**Table 2:** Total number of interactions detected by the Y2H screening after each filtering step.

Combination	Total	A + B Categories	No “Sticky” Prey	No Empty	No Duplicates
*pBC–pPC Library*	1093	355	31	25	15
*pBC–pPN Library*	1002	208	24	15	14
*pBN–pPC Library*	1448	458	119	119	106
*pBN–pPN Library*	934	122	54	52	47
**Total**	**4477**	**1143**	**228**	**211**	**182**

**Table 3:** Binary Y2H screening of 33 selected putative interactions between Bam35 (B35) baits and *B. thuringiensis* (Bt) preys using full-length proteins. The maximum 3AT concentration at which yeast growth was detected is gradually colored from red (low concentration) to green (high concentration). “No growth” indicates that no yeast growth was observed at any 3AT concentration.

Bait	Prey	Max. 3AT (mM)	Max. 3AT (mM) (Emptybait_BtORF)	Max. 3AT (mM)	Interaction	Bt Protein Coverage
(B35ORF)	(BtORF)	(B35ORF_Emptyprey)		(B35ORF_BtORF)		
pBC_06	pPC_pepA(24805)	0.1	50	0.1	N/A	0.33
pBC_10	pPC_dapF(25140)	50	10	10	N/A	0.44
pBC_25	pPC_hutI(18010)	0.025	50	No growth	No	0.43
pBC_25	pPC_pepA(24805)	0.025	50	0	N/A	0.33
pBC_26	pPN_pepD(12045)	No growth	25	No growth	No	0.37
pBC_31	pPC_hutI(18010)	0	50	0	N/A	0.43
pBC_31	pPC_pepA(24805)	0	50	0	N/A	0.33
pBC_31	pPC_tyrS(25855)	0	10	0	N/A	0.15
pBN_03	pPC_pbpX(02235)	No growth	0.1	No growth	No	0.44
pBN_06	pPC_pepA(24805)	No growth	0.1	No growth	No	0.33
pBN_08	pPN_lexA(18215)	No growth	0.1	No growth	No	0.42
pBN_11	pPC_pbpX(02235)	No growth	0.1	No growth	No	0.44
pBN_15	pPC_iap(27190)	No growth	0.1	25	Yes	0.76
pBN_15	pPN_(30985)	No growth	0.1	No growth	No	0.18
pBN_16	pPC_iap(27190)	No growth	0.1	25	Yes	0.76
pBN_16	pPN_(30985)	No growth	0.1	No growth	No	0.18
pBN_19	pPC_iap(27190)	No growth	0.1	50	Yes	0.76
pBN_19t	pPC_ompR(22050)	50	10	100	Yes	0.99
pBN_19t	pPN_menF(24455)	50	0.1	50	N/A	0.31
pBN_20	pPC_purL(01870)	No growth	0.1	No growth	No	0.24
pBN_22	pPC_yadS(28175)	0	0.1	50	Yes	0.74
pBN_22	pPN_(30985)	No growth	0.1	0	N/A	0.18
pBN_24	pPN_usp(26960)	0.025	0.1	0.1	N/A	0.54
pBN_25	pPC_hutI(18010)	No growth	0.1	No growth	No	0.43
pBN_25	pPC_pepA(24805)	No growth	0.1	No growth	No	0.33
pBN_25t	pPC_pepA(24805)	25	0.1	25	N/A	0.33
pBN_25t	pPN_pepA(24805)	25	0.1	25	N/A	0.33
pBN_26	pPC_(16200)	No growth	0.1	3	Yes	0.89
pBN_26	pPN_(30985)	No growth	0.1	No growth	No	0.18
pBN_27	pPC_iap(27190)	0.025	0.1	50	Yes	0.76
pBN_27	pPN_(30985)	No growth	0.1	No growth	No	0.18
pBN_27t	pPN_usp(26960)	3	0.1	0.1	N/A	0.54
pBN_31	pPC_hutI(18010)	0.1	0.1	No growth	No	0.43

**Table 4:** Putative PPIs between Bam35 (B35) proteins and *B. thuringiensis* (Bt) functional groups. ^a^ Truncated versions of proteins with a predicted transmembrane domain that had this domain removed are labeled with a “t.”

	B35 Protein (Function)	Bt COG ^b^ Interactions																				
		B	C	D	E	F	G	H	I	J	K	L	M	NO	O	P	Q	S	T	U	V	Total
Gene regulation and Genome replication	1 (DNA binding/phage cycle regulator *)									1												1
	2 (SSB)								1			1										2
	3 (Unknown)					1															1	2
	4 (TP)									1			1								1	3
	6 (Cycle regulator)		1		2	1						1				1		1				7
	7 (Cycle regulator)										1											1
	8 (Unknown)										1											1
Virion structure and DNA packaging	9 (Unknown)					1											1			1		3
	10 (DNA packaging *)		1		2													1				4
	11 (Assembly protein *)																	1			1	2
	12 (Unknown)																	1				1
	13 (Unknown)		1		3					1		1		1		2	1					10
	14 (DNA packaging/ATPase *)																	1				1
	15 (Special vertex *)		3	1			2					1		1	1				1			10
	16 (DNA packaging *)			1	1														1			3
	17 (Coat minor capsid protein *)		1			1			1				1			2						6
	19 (DNA packaging *)		3				1							1	1			3	1			10
	19t (DNA packaging *)				1			1				1						1	1			5
	20 (Stabilizer of spike *)				1	2	1		1				1			2		1				9
	20t (Stabilizer of spike *)						1											1			1	3
	21 (Unknown)				1		1	1														3
	22 (Special vertex *)		1	1			1									2		3				8
	23 (Unknown)		1				1								1	1		3	1		1	9
	24 (Penton *)		1					1					1		1	1	1					6
	25 (Membrane structural component)		1		3		1						1				1	2			2	11
	25t (Membrane structural component)				2		1	1	1	1												6
	26 (Transglycosylase and integral membrane scaffolding *)		2	1	1		2							1	1	2		5	2			17
	26t (Transglycosylase and integral membrane scaffolding *)	1	2		3	1	1	1		1						1	2	2				15
	27 (Unknown)		2	1			1							1	1			2	2			10
	27t (Unknown)												1									1
Host recognition and lysis	28 (Spike *)				1													1				2
	29 (Spike *)				1		1	1										1				4
	30 (Transglycosylase)				1		1															2
	31 (Unknown)				1					1							1	1				4
	Total	1	20	5	24	7	16	6	4	6	2	5	6	5	6	14	7	31	9	1	7	182

^a^ See Supplementary Table S6 for more detailed information. ^b^ **Cellular processes and signaling (pink):** (D) cell cycle control, cell division, chromosome partitioning; (M) cell wall/membrane/envelope biogenesis; (O) post-translational modification, protein turnover, and chaperones; (T) signal transduction mechanisms; (U) intracellular trafficking, secretion, and vesicular transport; (V) defense mechanisms. **Information storage and processing (green):** (**B**) chromatin structure and dynamics; (J) translation, ribosomal structure, and biogenesis; (K) transcription; (L) replication, recombination, and repair. **Metabolism (blue):** (C) energy production and conversion; (E) amino acid transport and metabolism; (F) nucleotide transport and metabolism; (G) carbohydrate transport and metabolism; (H) coenzyme transport and metabolism; (I) lipid transport and metabolism; (P) inorganic ion transport and metabolism; (Q) secondary metabolites biosynthesis, transport, and catabolism. **Limited characterization (gray):** (S) function unknown, (NO) no category assigned. * Suggested function.

## Data Availability

All sequencing data were deposited in the NCBI SRA database and is accessible via the BioProject PRJNA717632, or directly via the sample accession numbers listed in Supplementary Table S2. The Bam35–*B. thuringiensis* high-quality interactions reported in Supplementary Table S6 were also deposited in the Database of Interacting Proteins under the IMEx Consortium dataset identifier IM-28933.

## Supplementary Materials

The following are available online at https://www.mdpi.com/article/10.3390/ijms222011105/s1.
