# Supplementary material for: Unraveling Protein Interactions between the Temperate Virus Bam35 and Its Bacillus Host Using an Integrative Yeast Two Hybrid–High Throughput Sequencing Approach
Source: Int J Mol Sci. 2021 Oct 14;22(20):11105. doi: 10.3390/ijms222011105 (PMC8539640; doi:10.3390/ijms222011105)
Supplement: Supplementary file 1 [file ijms-22-11105-s001.zip › Supporting_information_IJMS_revised_TBS.pdf]

SUPPORTING INFORMATION

UNRAVELING PROTEIN INTERACTIONS BETWEEN THE TEMPERATE VIRUS BAM35 AND ITS *BACILLUS* HOST USING AN INTEGRATIVE YEAST TWO HYBRID-HIGH THROUGHPUT SEQUENCING APPROACH

Ana Lechuga[1,2], Cédric Lood[2,3 #], Mónica Berjón-Otero[1 #], Alicia del Prado[1], Jeroen Wagemans[2], Vera van Noort[3], Rob Lavigne[2], Margarita Salas[1 ‡] and Modesto Redrejo-Rodríguez[1,4 *]

[1]Centro de Biología Molecular Severo Ochoa (CSIC-UAM) Madrid, Spain.

[2]Department of Biosystems, Laboratory of Gene Technology, KU Leuven, Leuven, Belgium

[3]Department of Microbial and Molecular Systems, Centre of Microbial and Plant Genetics, Laboratory of Computational Systems Biology, KU Leuven, Leuven, Belgium

[4]Departamento de Bioquímica, Universidad Autónoma de Madrid (UAM) and Instituto de Investigaciones Biomédicas Alberto Sols (CSIC-UAM), Madrid, Spain.

[#]Contributed equally to this work.

[‡]Deceased.

*Correspondence to Modesto Redrejo Rodríguez

Departamento de Bioquímica

School of Medicine (Universidad Autónoma de Madrid)

Arzobispo Morcillo, 4

28029 – Madrid (SPAIN)

Telephone: +34 91 497 5963

E-mail: modesto.redrejo@uam.es

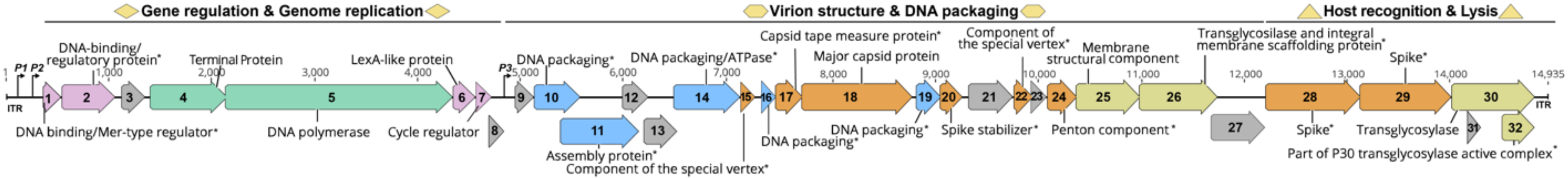

Supplementary Figure S1. **Genetic map of the Bam35 genome**. Predicted coding sequences (CDSs) are indicated with arrows of proportional width. Suggested (*) or known functions are indicated and grouped by color: regulatory elements (pink), replication (green), assembly and DNA packaging (blue), capsid structure component (orange), membrane structural component and lysis (yellow). Unknown function CDSs are colored in grey. Position and direction of viral promoters *P1*, *P2* and *P3* are marked by angled arrows and inverted terminal repeats (ITRs) are also indicated. The rulers represent base pairs. Figure adapted from Gillis et al. 2018 [1].

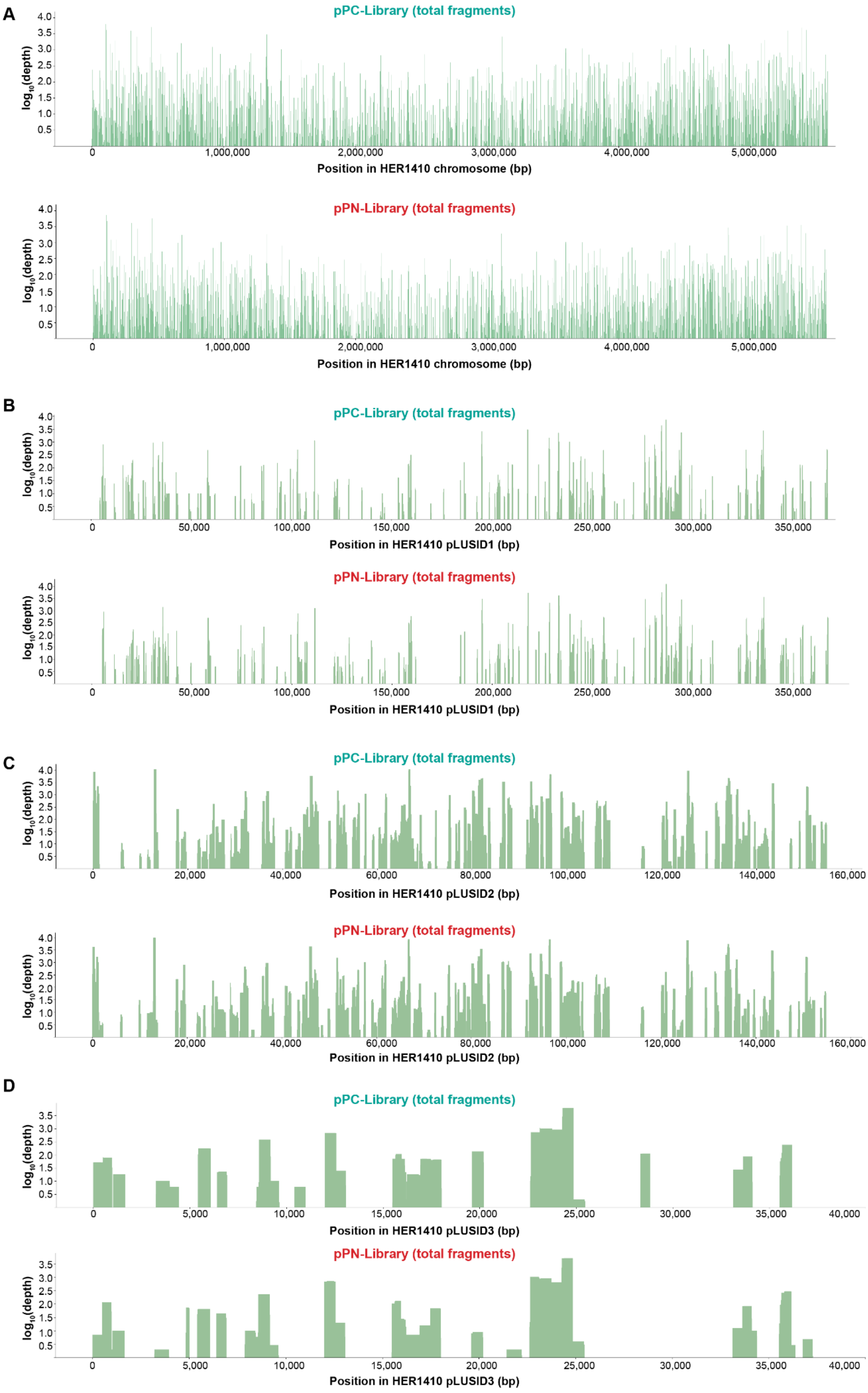

Supplementary Figure S2. Coverage graphics of HER1410 libraries. Depth coverage of raw fragments of pPC or pPN libraries in the HER1410 chromosome **(A)** and plasmids **(B, C and D)** (GenBank IDs. CP050183-6). In the graphics, coverage is shown in the log scale (Y-axis) to display both highly and little covered positions in the HER1410 chromosome (X-axis).

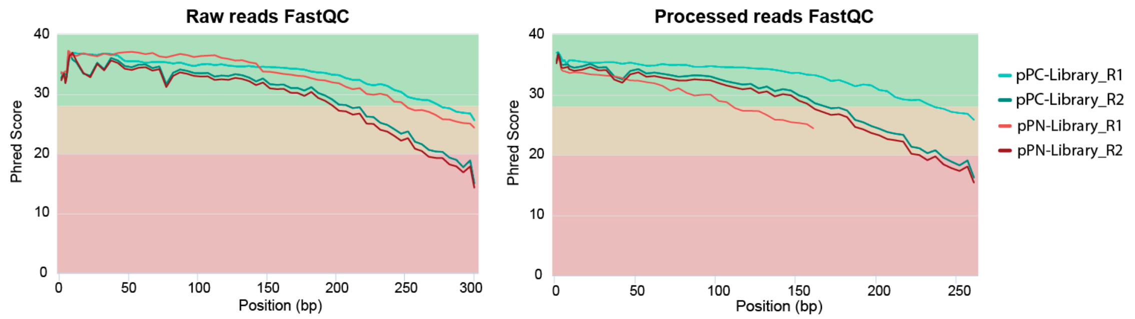

Supplementary Figure S3. Quality control of the raw and processed reads of pPC (tile) and pPN (red). The Illumina paired-end reads (R1 and R2, forward and reverse, respectively) were verified for quality using FastQC before (raw reads) and after (processed reads) trimming using seqtk and Trimmomatic. FastQC reports of raw and processed reads were consolidated and visualized using MultiQC. The Y-axis represents the average Phred quality score and the X-axis represents the position within the Illumina reads.

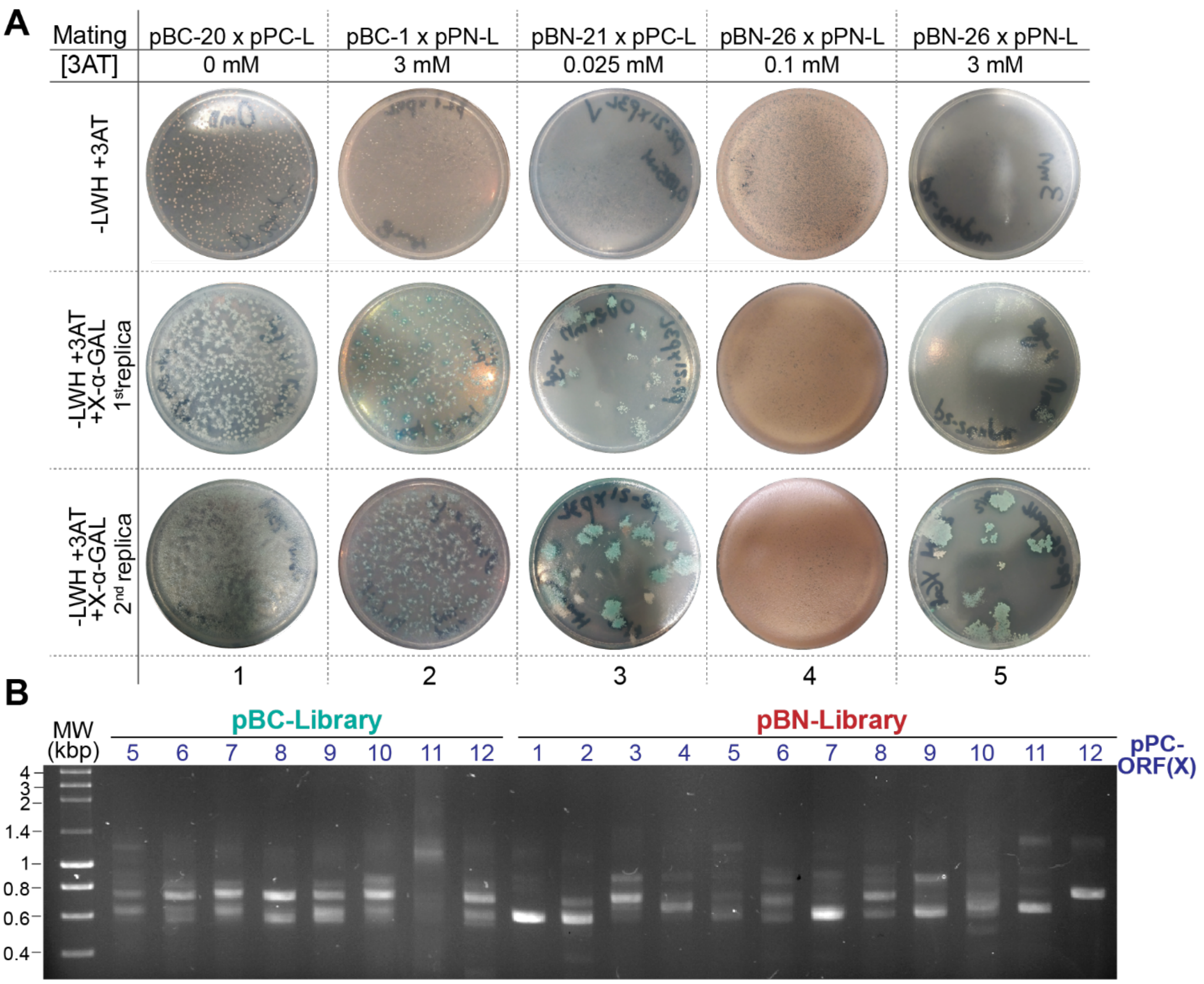

Supplementary Figure S4. Representative output of the Bam35-*B. thuringiensis* Y2H screen. (A) Representative pictures of the three-step plating of four different bait-prey combinations. Plates from columns 4 and 5 correspond to the same combination with different 3AT concentration. Cells harboring interacting partners are able to grow in the absence of histidine, and therefore on -LWH media. Strong interactions were detected as blue colonies as a result of the metabolism of X-α-Gal in the first and second replicas. pBC-X and pBN-X stand for pBC and pBN harboring the corresponding viral ORF (X) and pPC-L and pPN-L stand for pPC and pPN HER1410 libraries respectively. **(B)** Representative gel of the prey inserts PCR- amplification from different bait-prey matings. Cells from the last replica were harvested, their plasmid DNA was extracted, and the prey fragments were PCR amplified as explained in Materials and Methods to avoid amplification bias. For each sample/assay, several bands were detected whose size range was in agreement with the libraries size range.

pB-B35ORF	pP-BtORF	pB-empty x pP-BtORF	pB-B35ORF x pP-empty	pB-B35ORF x pP-BtORF	3AT
pBN-26<br>Transglycosylase	pPC-16200<br>Hypothetical protein	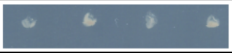	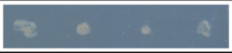	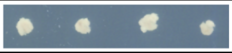	3mM
pBN-15<br>Special vertex *	pPC-27190<br>SH3 domain-containing protein	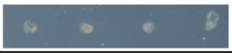	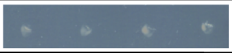	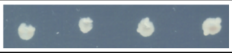	25mM
pBN-16<br>DNA packaging*	pPC-27190<br>SH3 domain-containing protein	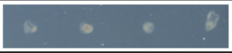	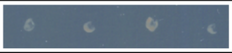	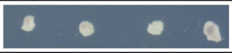	25mM
pBN-19<br>DNA packaging*	pPC-27190<br>SH3 domain-containing protein	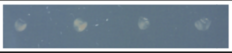	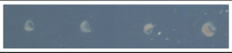	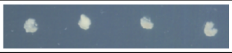	50mM
pBN-27<br>Pentameric base spike*	pPC-27190<br>SH3 domain-containing protein	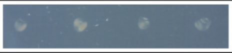	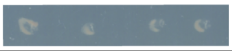	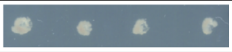	50mM
pBN-22<br>Special vertex*	pPC-28175<br>Trimeric intracellular cation channel protein	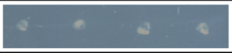	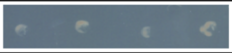	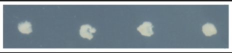	50mM
pBN-19t<br>DNA packaging*	pPC-22050<br>Response regulator transcription factor	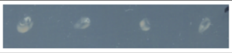	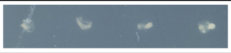	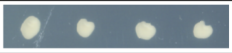	100mM

Supplementary Figure S 5. Confirmed interactions between full-length proteins by binary Y2H. Retesting of 33 potential interactions was carried out by pairwise Y2H screening at different 3AT concentrations (see Methods). In this case, the complete host gene was cloned into the correspondent prey vector and assayed with its bait partner according to Table 3. A total of seven positive interactions were validated and are shown in the figure. Interactions were considered positives when the growth of diploids containing pB-X and pP-Y, being X the Bam35 (B35) ORF and Y the *Bacillus thuringiensis* HER1410 (Bt) locus, was higher than the combinations containing pB-empty or pP-empty. The images in all the panels correspond to those obtained with the highest 3AT concentration at which the interaction was detected.

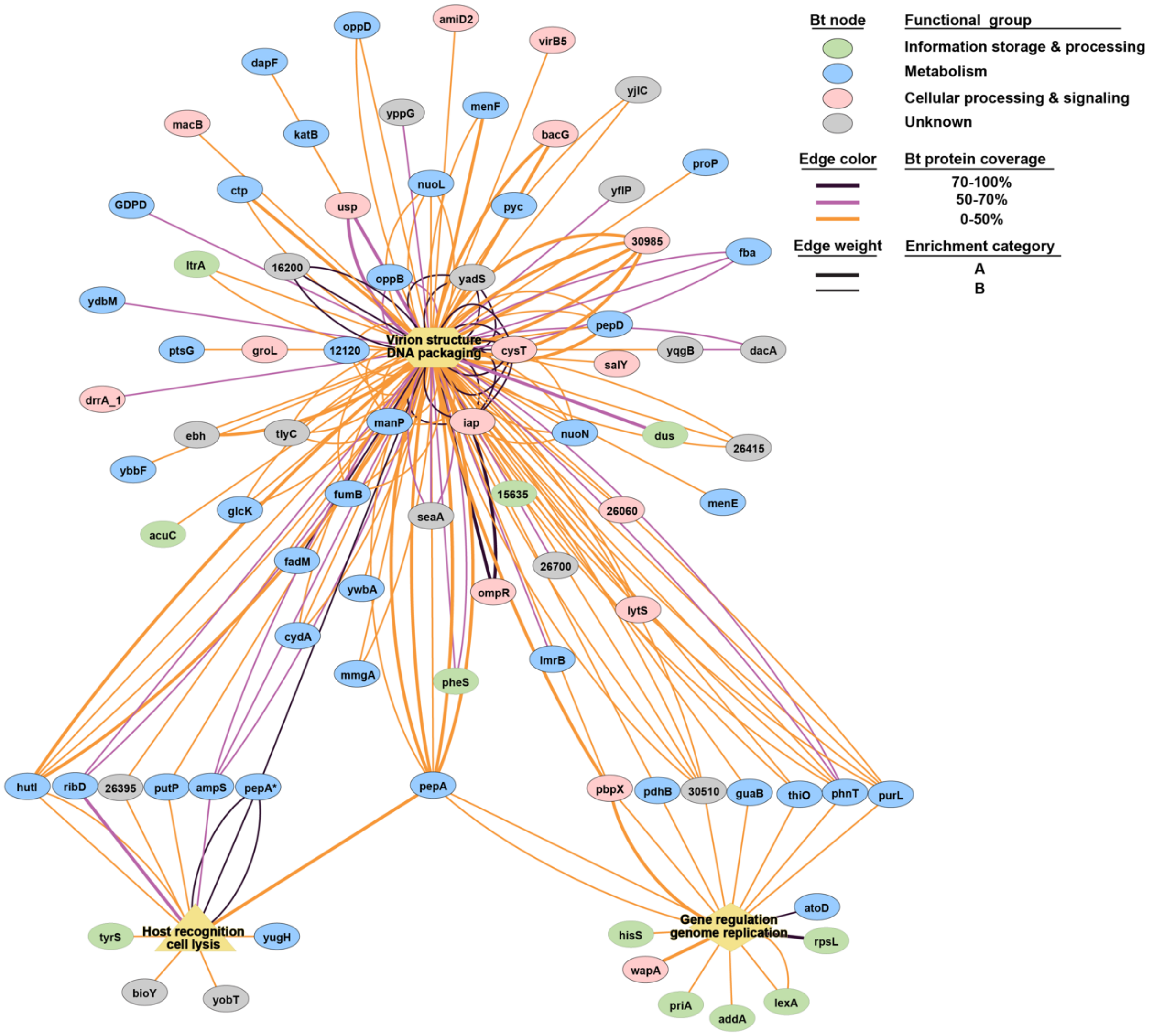

Supplementary Figure S6. Detected interactions between *B. thuringiensis* proteins and functional groups of Bam35 proteins detected in our screen. Network of the 182 detected putative interactions between the host proteins and the viral proteins grouped by function (big yellow nodes), detailed in Supplementary Table 6. Host proteins (Bt nodes) are colored according to their functional groups. Host proteins are identified by their annotated function or, for the unknown function proteins, their locus number in HER1410 genome (accession numbers available in Supplementary Table 6). Each interaction is represented as a line connecting two nodes (edge). Line colors indicate the length coverage of the host protein by the detected prey fragment and line weight indicates the enrichment category of the interaction (B for prey proteins detected as 0.25-10% of the sample counts and A for 10-100%). PepA* refers to the M42 family metallopeptidase encoded by the host gene HBA75_23575 and PepA refers to the cytosol aminopeptidase encoded by the host gene HBA75_24805.

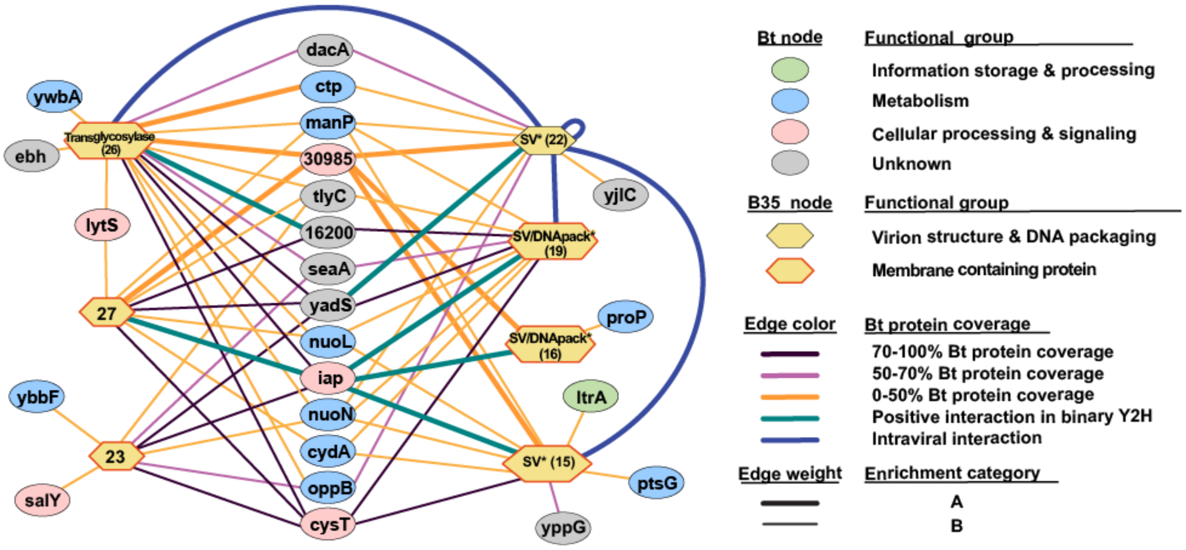

Supplementary Figure S7. Differentiated cluster in the Bam35-*B. thuringiensis* putative interactome (Figure 5). This cluster shows a group of Bam35 proteins with unknown and related functions (Special vertex and DNA delivery) interconnected by a high number of host proteins. The intraviral interactions described in Berjón-Otero *et al.* [2] (thick blue edges) and the interactions confirmed by pairwise Y2H assays (thick green edges) are shown.

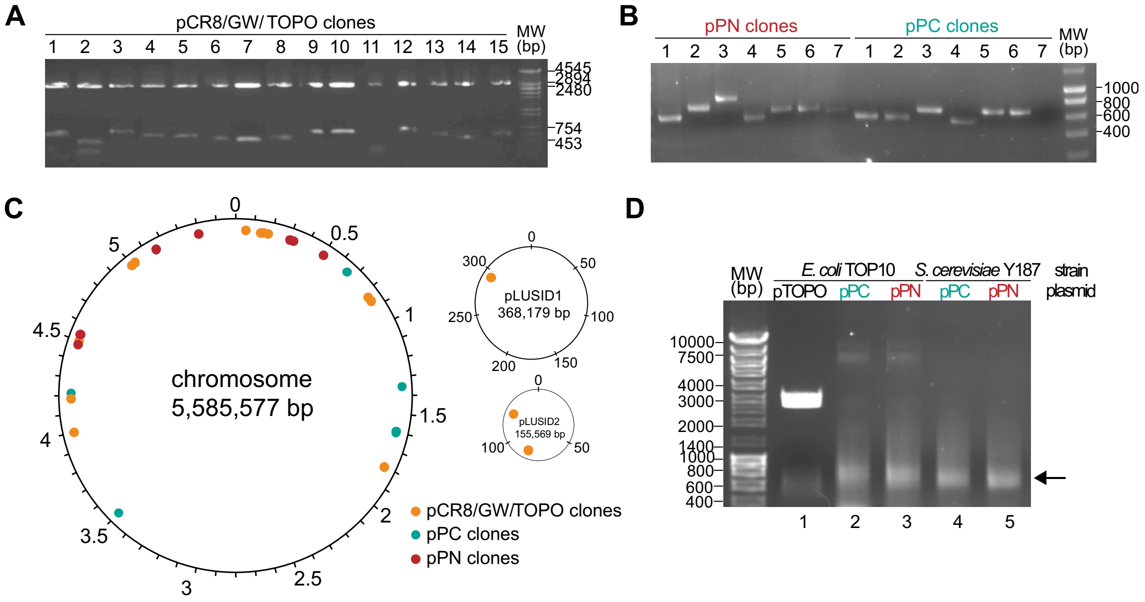

Supplementary Figure S8. Preliminar analysis of the fragment-based HER1410 libraries. (A) *EcoRI* restriction analysis of 15 independent clones from the HER1410 genomic library in pCR8/GW/TOPO. The library inserts size range (453-754 bp) corresponds to the expected size. **(B)** PCR amplification of library inserts from 14 independent clones from the genomic libraries in pPC and pPN **(C)** Sequencing of the individual library inserts in (A) and (B). The components of the HER1410 genome (GeneBank: CP050183-CP050186S) are represented and the location of the analyzed inserts is shown as orange (pCR8/GW/TOPO), teal (pPC), and red (pPN) spots. **(D)** Genomic libraries analysis by *EcoRI* restriction analysis (lane 1) and PCR amplification (lanes 2-5). The library inserts DNA is indicated by an arrow. The library inserts size range (453-754 bp) corresponds to the expected size.

Supplementary text

Data analysis reveals a good performance of the Y2H-HTS approach

The PCR products from the Y2H assays were sequenced in an Illumina MiSeq 300-bp paired-end run, obtaining 12,229 reads on average per screen/sample (Supplementary Table S2). For some samples, secondary PCR product analysis revealed the presence of fragments shorter than 300 bp which were separated for independent sequencing (<300 bp and >300bp) to reduce sequencing bias. The Illumina reads were processed using the data filters previously explained (Figure S 1) and the detailed filtering results are shown in Supplementary Figure S 8 and Supplementary Table S2. This filtering process yielded 28% of the total reads as Y2H-validated fragments (Supplementary Figure 8A). As expected, a high proportion (78%) of fragments under 300 bp did not pass the size and "ATG" filters due to their size. In turn, about 60% of the fragments over 300 bp passed all filters. This contrasts with the results of filtering the HER1410 libraries reads where only 1-2% of the reads correspond to fragments validated for Y2H. This shows a good performance of the Y2H technique, which appears to strongly favor those fragments in frame with the activation domain and the actual ORFs within the genome, with only minor library-borne background. When the samples including pPC or pPN libraries were compared, significant differences were observed in terms of final validated fragments (Supplementary Figure 8B). Thus, the combination with the pPN library would lead to a higher non Y2H specific background. Finally, after data treatment, mapping of the Y2H-validated fragments allowed us to retrieve a total of 4,477 possible interactions (Supplementary Table S3).

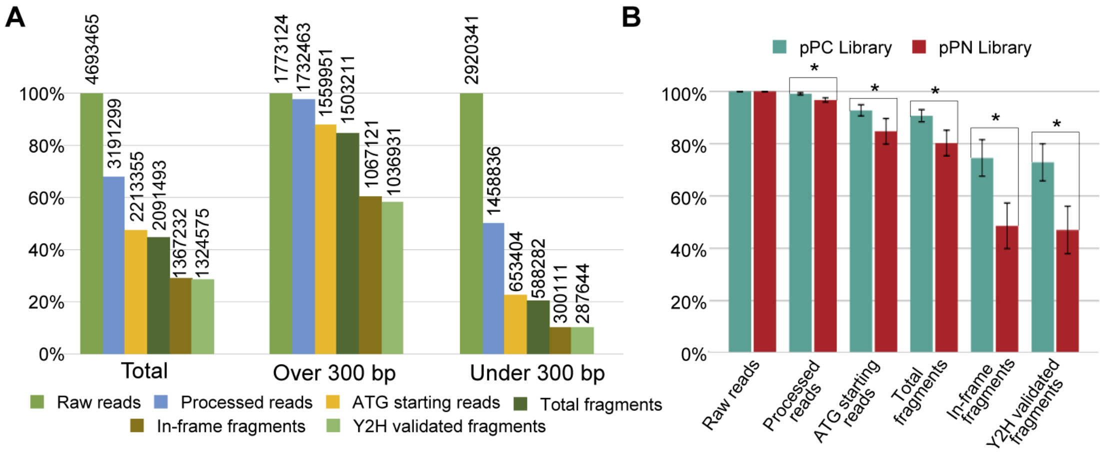

Supplementary Figure S9. Filtering of the Y2H positives sequencing data. (A) Bar plot of the proportion of reads/fragments kept after each of the filters applied to the sequencing data from the Y2H screen (see Figure 2). The corresponding number of reads/fragments is indicated above each bar. For technical purposes, the fragments were sequenced in two different runs regarding their size, over or under 300 bp, indicated in the figure. **(B)** Over 300 bp dataset comparison between the pPC and pPN libraries. The comparison of the libraries was assessed with a Krustal-Wallis test for independent samples using SPSS® Statistics software (IBM). Statistic differences ($p<0.05$) between libraries are indicated by a star.

A correlation test between the enrichment, i.e. the normalized read counts, of library fragments and their presence in the Y2H positive results was performed for the two libraries. In both cases, the distribution of hits did not show any discernable pattern and the correlation coefficient was not significantly different from 0 ($p<0.01$) (Supplementary Figure S9). Thus, we can conclude that,

in this experimental setup, an enrichment in the library is not linked with the presence in the Y2H positives. This could also indicate a minor contribution of a putative library-originated background.

Interestingly, correlation analyses revealed a high enrichment of hits in the 0-5% range of normalized read counts.

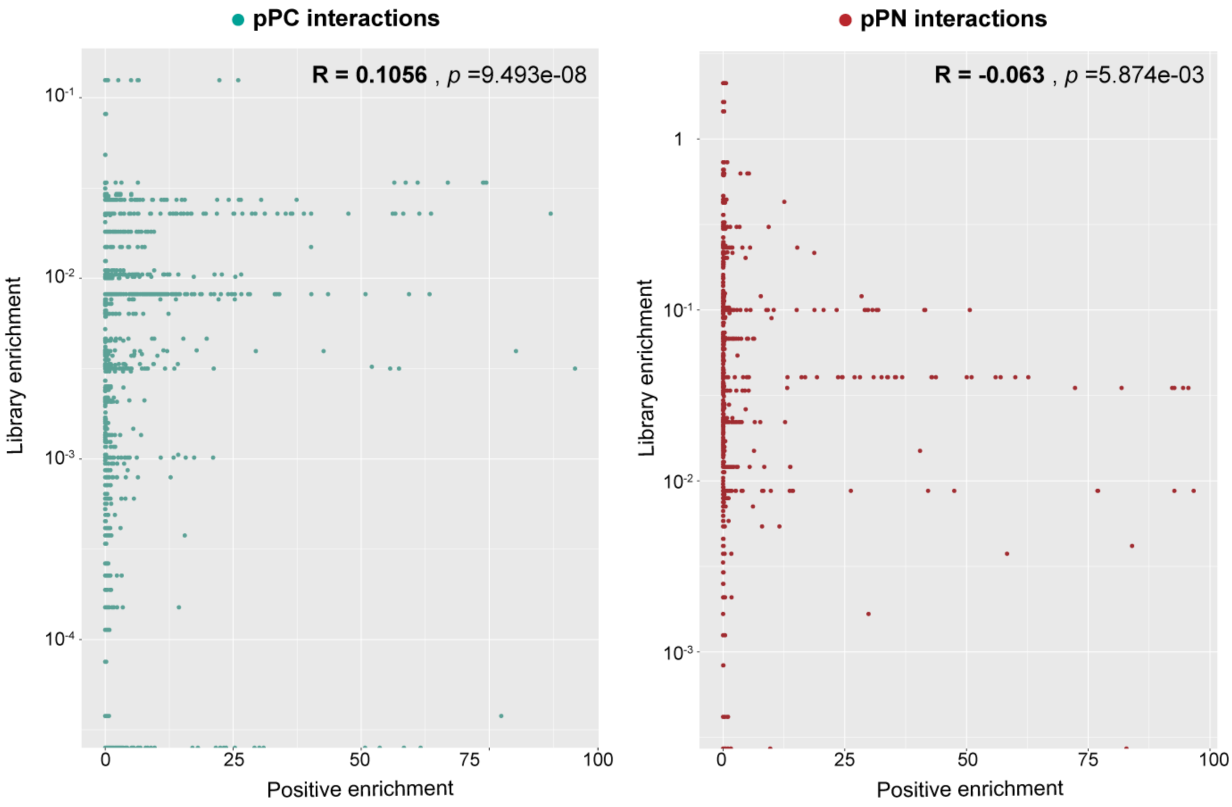

Supplementary Figure S10. Correlation between library enrichment and Y2H positives enrichment values. Scatterplot of the enrichment (normalized read counts value) of the prey loci detected in the Y2H screens (X-axis) and their enrichment in the corresponding library (Y-axis). Correlation coefficients and *p*-values are shown.

The distribution of the normalized read counts showed that 75% of the hits had an enrichment below 0.26% (Supplementary Figure S 10) meaning that 75% of the total interactions were represented by less than 0.26% of the reads of the corresponding sample. To remove the sequencing data background and organize the interactions according to their presence in the

dataset, we decided to use the enrichment value to establish three enrichment categories as follows: A (100-10% of normalized counts) B (10%-0.25%), and C (0.25%-0%). Although a total of 3,334 interactions out of 4,477 were grouped in the category C, they correspond to only 2.04% of the total reads (Table S1). As shown in Supplementary Table S8, if we analyze the interactions before the last filtering step (AD frame validation in Figure S1), most of the interactions whose

enrichment value would correspond to the categories A and B included fragments in frame with the AD domain. This is in agreement with the key role of this domain in the interaction detection. In contrast, the number of fragments from the category C interactions were equally distributed between in-frame and not in-frame inserts. This suggests that interactions falling into this category

cannot be properly distinguished from the non-relevant background and, therefore, linked to a relevant Y2H result. This, together with the fact that numerous category C interactions represent a low percentage of the total data and fall into the region in Supplementary Figure S10 probably linked to the background noise due to deep sequencing analysis, we decided to take them apart from the main dataset.

Percentiles	5	10	25	50	75	90	95
Weighted Average	0.00213	0.00508	0.00853	0.032922	0.26201	3.51608	10.71126

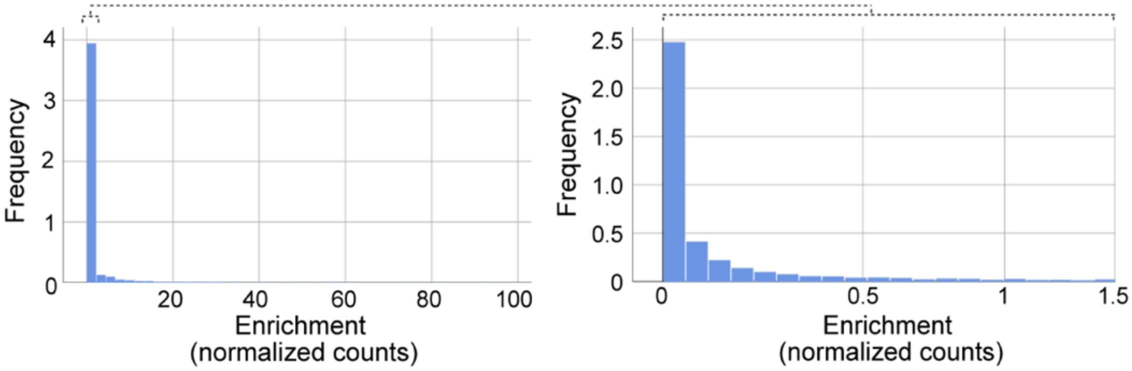

Supplementary Figure S 11. Y2H hits distribution based on their enrichment value. Frequency distribution analysis for the normalized read counts (enrichment) detected in the Y2H screen. Percentiles (upper panel) and histograms (lower panel) obtained with SPSS® Statistics software (IBM) are shown. On the right, a subset of the data corresponding to the 0-1.5 normalized counts region is shown. Here, a base-10 log scale was used for the X-axis.

Also, a small-scale sequencing analysis was performed to validate the high-throughput sequencing results. Before harvesting the cells, a total of 70 randomly picked colonies were isolated from the last replica plates of the Y2H screen and their prey inserts were amplified and identified by colony PCR and Sanger sequencing (Supplementary Table S9). Here, consistently with previous results, about 70% of the prey fragments were in frame with the AD domain

(Supplementary Figure S11). The identified in-frame fragments were then searched into the Ilumina sequencing dataset. All of them but eight interactions were found. Interestingly, the eight missing interactors are encoded by IS*4* family transposase genes. IS transposases appear to be “sticky”

preys (see below) and their sequence is highly repetitive and variable, hampering the mapping of the Sanger sequencing reads to the same exact *B. thuringiensis* genomic locus in the sequencing

dataset.

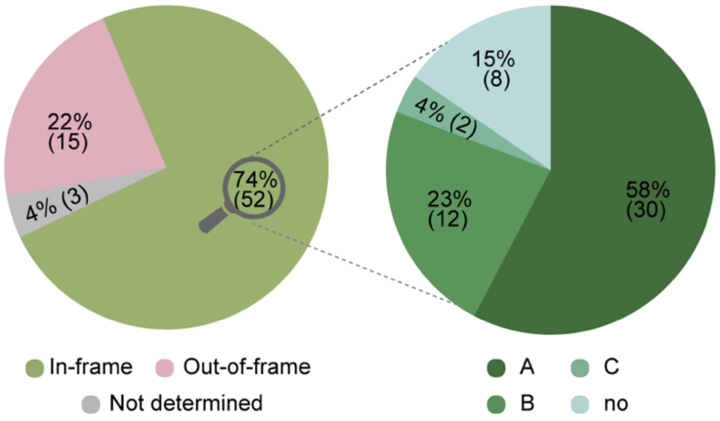

Supplementary Figure S 12. Individual Sanger sequencing of Y2H positive colonies. A total of 70 randomly picked colonies from different assays were analyzed by colony PCR amplification and Sanger sequencing of the prey fragment (Supplementary Table 9). The insert reading frame was determined for each fragment (left). Those whose low-quality sequence hampered classification are indicated as not determined. For those sequences that were in frame with the *AD* gene, their enrichment category in the high-throughput sequencing dataset was determined (right). The sequences that were in frame but could not be found in the sequencing dataset are indicated in light green (marked as “no”).

The rest of the interactions were readily linked to the Illumina seq-detected interactions and their corresponding categories. In agreement with the enrichment values and designed categories, most of the sequences corresponded to A and B category interactions, with only two sequences linked to the C category. Also, all but one of the interactions detected more than once by Sanger

were classified in the Illumina dataset as category A. Overall, these results confirmed the good performance of our pipeline in which Y2H interactions are identified through high-throughput sequencing and classified according to the normalized read counts and the enrichment categories. Besides, the identification of all the manually isolated Y2H positives in the Illumina dataset indicates a high rate of positive recovery through this sequencing approach. On the other

hand, no enrichment differences were observed between white and blue colonies (Supplementary Table S9) and the prey fragments identified in blue colonies often corresponded to “sticky” preys or false positives (see Materials and Methods). Therefore, the *MEL1* reporter was not further used for the analysis of the Y2H results since it did not provide relevant information to this study.

References

1. Gillis A, Fayad N, Makart L, Bolotin A, Sorokin A, Kallassy M, et al. Role of plasmid plasticity and mobile genetic elements in the entomopathogen Bacillus thuringiensis serovar israelensis. FEMS Microbiol Rev. 2018;42: 829–856. doi:10.1093/femsre/fuy034

2. Berjón-Otero M, Lechuga A, Mehla J, Uetz P, Salas M, Redrejo-Rodríguez M. Bam35 tectivirus intraviral interaction map unveils new function and localization of phage ORFan proteins. J Virol. 2017;91. doi:10.1128/JVI.00870-17
